# Glycerol as Precursor of Organoselanyl and Organotellanyl Alkynes

**DOI:** 10.3390/molecules22030391

**Published:** 2017-03-02

**Authors:** Eder J. Lenardão, Elton L. Borges, Guilherme Stach, Liane K. Soares, Diego Alves, Ricardo F. Schumacher, Luana Bagnoli, Francesca Marini, Gelson Perin

**Affiliations:** 1 Laboratório de Síntese Orgânica Limpa (LASOL), Centro de Ciências Químicas, Farmacêuticas e de Alimentos (CCQFA), Universidade Federal de Pelotas (UFPel), P.O. Box 354, 96010-900 Pelotas, RS, Brazil; chemistry_borges@yahoo.com.br (E.L.B.); guilherme.stach13@gmail.com (G.S.); lianekrolowsoares@yahoo.com.br (L.K.S.); dsalves@gmail.com (D.A.); ricardo.schumacher@ufpel.edu.br (R.F.S.); 2 Department of Pharmaceutical Sciences, Group of Catalysis and Organic Green Chemistry, University of Perugia, Via del Liceo 1, 06100 Perugia, Italy; luana.bagnoli@unipg.it (L.B.); francesca.marin i@unipg.it (F.M.)

**Keywords:** selenium, tellurium, 1,3-dioxolanes, glycerol, alkynes

## Abstract

Herein we describe the synthesis of organoselanyl and organotellanyl alkynes by the addition of lithium alkynylchalcogenolate (Se and Te) to tosyl solketal, easily obtained from glycerol. The alkynylchalcogenolate anions were generated in situ and added to tosyl solketal in short reaction times, furnishing in all cases the respective products of substitution in good yields. Some of the prepared compounds were deprotected using an acidic resin to afford new water-soluble 3-organotellanylpropane-1,2-diols. The synthetic versatility of the new chalcogenyl alkynes was demonstrated in the iodocyclization of 2,2-dimethyl-1,3-dioxolanylmethyl(2-methoxyphenylethynyl)selane **3f**, which afforded 3-iodo-2-(2,2-dimethyl-1,3-dioxolanylmethyl) selenanylbenzo[*b*]furan in 85% yield, opening a new way to access water-soluble Se-functionalized benzo[*b*]furanes.

## 1. Introduction

Organochalcogen chemistry is considered a very broad field due to the increasing research on the synthesis [1,2,3,4,5] and applications [2,6,7,8,9] of this class of compounds. Besides, organoselanyl and organotellanyl alkynes have become extensively studied due their pharmacological and biological activities [10,11,12,13] and their use as starting material in organic synthesis [2,14,15,16]. For example, organotellanyl alkynes exhibited antidepressive-like activity [17], while alkyne-derived organotellanyl alkenes showed in vitro antioxidant activity with slight toxicity [18,19]. Additionally, chalcogenyl alkynes are useful in electrophilic cyclization reactions to prepare benzo[*b*]furans [20], in [4 + 2]-cycloadditions to produce the corresponding 2-chalcogenyl-1-halonaphthalenes [21], and in the synthesis of bis-phenylchalcogen alkenes [22,23]. Despite all the advances in this area, most of the papers are restricted to the synthesis of chalcogenyl alkynes starting mainly from aromatic terminal alkynes [24,25,26,27,28,29,30], with only a few methods to prepare aliphatic alkynylselenides and tellurides with different chalcogen substitution patterns.

On the other hand, with the increasing overproduction of glycerol [31], solketal has become a useful intermediate in chemical transformations using glycerol as a raw material. It is well accepted that tosyl solketal plays an important role in a vast array of applications [32,33,34,35,36]; standing out is its use as a building block in organic synthesis. Recently, tosyl solketal was used as starting material for the synthesis of several biologically active compounds [36,37]. The synthesis of a series of organochalcogen glycerol derivatives was described, including chalcogen ethers [38,39] with antioxidant activity [38], as well as enantiomerically pure selenides and diselenides [40].

**Scheme 1:**
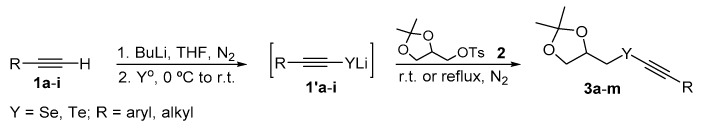
General scheme of the reaction.

In a previous paper [41], we described a convenient procedure for the synthesis of new vinyl chalcogenides by the reaction of glycerol-derived dichalcogenides with terminal alkynes in the presence of NaBH_4_, using PEG-400 as the solvent. Chalcogenyl alkynes were selectively prepared from the same starting materials, when ethanol was the solvent. However, reaction times were in the range of 5 to 26 h, and the scope of the reaction was limited to organoselanyl alkynes, since the synthesis of only one organotellanyl alkyne in 55% yield was reported [41]. Trying to solve these limitations, and in continuation of our studies on the synthesis and reactivity of chalcogenyl alkynes, herein we describe a general and efficient synthesis of a new glycerol-derived organoselanyl and organotellanyl alkyne **3** via the nucleophilic substitution of lithium alkynylchalcogenolate **1′** (Se and Te) with tosyl solketal **2** (Scheme 1).

## 2. Results and Discussion

The first step of the reaction is the preparation of the nucleophilic species **1′a**–**i**, which were prepared in situ using a butyllithium solution and THF as the solvent. Phenylacetylene **1a** (1.0 mmol) and elemental selenium (1.0 mmol) were used as standard reagents to optimize the preparation of the respective alkynylselenolate **1′a** (R = C_6_H_5_; Y = Se) at 0 °C under N_2_ atmosphere. After stirring for 20 min at 0 °C, racemic tosyl solketal **2** (1.2 mmol) was added and the mixture was stirred at room temperature for an additional 1 h, giving 2,2-dimethyl-1,3-dioxolanylmethyl(phenylethynyl)selane **3a** in 20% yield. When the reaction time was extended to 3 h, the yield was increased to 30%, but side products were also observed. Next, the amount of tosyl solketal **2** was reduced to 1.0 mmol (1 equiv. related to the alkynylselenolate anion **1′a**) and after 3 h at r.t., **3a** was isolated in 50% yield. However, the best performance of this reaction was achieved when the amount of tosyl solketal **2** was decreased to 0.5 mmol (0.5 equiv. related to **1′a**), affording **3a** in 80% yield after 3 h. By using 0.7 equiv. of tosyl solketal **2**, a decrease in the yield of product **3a** was observed (42%). These findings indicate that a large excess of chalcogenolate anion is mandatory for the reaction.

After determining the best conditions to prepare **3a**, the protocol was extended to the differently substituted aliphatic and aromatic terminal alkynes **1b**–**h** (Table 1, entries 2–8). As shown in Table 1, a number of selenanyl alkynes were prepared in good yields. Starting from aliphatic hex-1-yne **1b**, 2,2-dimethyl-1,3-dioxolanylmethyl(hex-1-yn-1-yl)selane **3b** was obtained in 63% yield. This is a good outcome, considering that an aliphatic alkyne was used as the starting material (Table 1, entry 2). In principle, the presence of substituents in the aromatic ring of alkynes **1c**–**g** seems to negatively impact the reactivity, since all the respective products were obtained in lower yields. Experiments have shown that the overall result is similar in both situations; i.e., the yields of products **3c**–**g** are reduced to 60%–70% (Table 1, entries 3–7). Ethynylcyclohex-1-ene **1h** afforded the respective selenanyl alkyne **3h** in 52% yield, showing that the reaction can be successfully applied to conjugated enynes (Table 1, entry 8).

The optimized protocol was employed using tellurium instead selenium, in order to prepare organotellanyl alkynes **3i**–**m**. However, by reacting phenylacetylene **1a** with elemental tellurium under the same reaction conditions employed for the selenium derivatives, the respective 2,2-dimethyl-1,3-dioxolanylmethyl(phenylethynyl)tellane **3i** was obtained in only 30% yield after 3 h. Trying to improve the yield of **3i**, the same procedure was repeated, but the tosyl solketal **2** was added at 0 °C and the temperature maintained at 0 °C for an additional 3 h. In this case, the desired product **3h** was obtained only in trace amounts, with a large amount of the bis(phenylethynyl)tellane as side product. Then, an additional test was performed: after addition of the solketal **2** to the previously formed alkynyltellurolate **1′** at 0 °C, the ice bath was replaced by an oil bath and the reaction mixture was stirred under reflux for 1.5 h. To our delight, this reaction afforded the desired tellanyl alkyne **3i** in 85% yield (Table 1, entry 9). Under the new conditions, the reaction scope could be expanded to other alkynes and the respective tellanyl alkynes were obtained in good yields, similar to the selanyl alkynes analogues (Table 1, entries 10–13).

Due to our interest in the synthetic applications of organochalcogen compounds, as well as in studying new possibilities in their biological activities, we performed the synthesis of new 3-organotellanylpropane-1,2-diols **4** (Table 2). Starting from the organotellanyl alkynes **3i**, **3l**, and **3m**, the 3-tellanylpropane-1,2-diols **4a**–**c** were obtained in moderate yields by treatment with Dowex 50WX8 (H^+^ form) in methanol, after 5 h (Table 2, entries 1–3). These new compounds generally showed a good solubility in water, which will facilitate biological tests where aqueous medium is required (Table 2).

**Scheme 2:**
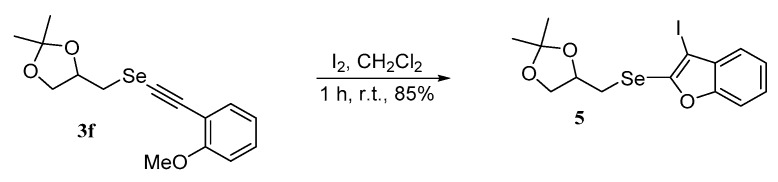
Synthesis of 3-iodo-2-(2,2-dimethyl-1,3-dioxolanylmethyl)selenanylbenzo[*b*]furan **5**.

Selanyl alkynes with an appropriate substitution pattern are attractive intermediates in organic synthesis [20]. For example, the intramolecular electrophilic cyclization of **3f** with iodine was easily conduced in CH_2_Cl_2_ at r.t. for 1 h, delivering 85% yield of the densely functionalized 3-iodo-2-(2,2-dimethyl-1,3-dioxolanylmethyl)selenanylbenzo[*b*]furan **5** (Scheme 2). The combination of a selenium group with the benzo[*b*]furan scaffold could be an interesting strategy in the prospection of new drug candidates.

## 3. Experimental Section

### 3.1. General Information

The reactions were monitored by thin layer chromatography (TLC) carried out on Merck (Merck, Darmstadt, Germany) silica gel (60 F_254_) by using UV light as visualizing agent and 5% vanillin in 10% H_2_SO_4_ and heat as developing agent. NMR spectra were recorded with Bruker spectrometer (Bruker, Billerica, MA, USA) DPX 300, DPX 400, and DPX 500 (300, 400, and 500 MHz, respectively) instruments using CDCl_3_ as solvent and calibrated using tetramethylsilane (TMS) as internal standard. Chemical shifts (δ) are reported in ppm, coupling constants (*J*) are reported in Hertz. The NMR spectra are found in the Supplementary Materials. Low-resolution mass spectra were obtained with a Shimadzu GC-MS-QP2010 mass spectrometer (Shimadzu Corporation, Kyoto, Japan) and molecular ion values are reported according the exact mass. High-resolution mass spectra (HMRS) were recorded on a Shimadzu LC-MS-IT-TOF spectrometer (Shimadzu Corporation). Melting points were determined using a Marte PFD III melting point instrument (Marte Científica, Minas Gerais, Brazil).

### 3.2. General Procedure for Synthesis of Organoselanyl Alkynes ***3a***–***h***

To a solution of alkyne **1** (1.0 mmol) in THF (5.0 mL) under N_2_ atmosphere, BuLi (1.6 mol/L in hexanes; 1.0 mmol) was added at 0 °C. After 20 min, the temperature was increased to room temperature, and elemental selenium (Se^0^, 1.0 mmol) was added. The stirring at room temperature was maintained until all selenium was consumed, and then a solution of racemic tosyl solketal **2** (0.5 mmol) in THF (2.0 mL) was added. After stirring for 3 h, the reaction mixture was quenched with water (15.0 mL) and extracted with ethyl acetate (3 × 15.0 mL). The organic phase was separated, dried over MgSO_4_, and the solvent was evaporated under reduced pressure. The product was isolated by column chromatography using hexanes/ethyl acetate as eluent.

#### Analytical Data of Products **3a**–**h**

*2,2-Dimethyl-1,3-dioxolanylmethyl(phenylethynyl)selane* **3a** (Table 1, entry 1) [41]: Yield: 0.118 g (80%); yellow oil. ^1^H-NMR (CDCl_3_, 300 MHz); δ (ppm): 7.38–7.42 (m, 2H, Ar-H), 7.27–7.32 (m, 3H, Ar-H), 4.46–4.54 (m, 1H, O-CH), 4.21 (dd, *J* = 8.6 and 6.0 Hz, 1H, O-HCH), 3.88 (dd, *J* = 8.6 and 5.8 Hz, 1H, O-HCH), 3.07 (dd, *J* = 12.1 and 5.2 Hz, 1H, Se-HCH), 2.94 (dd, *J* = 12.1 and 7.7 Hz, 1H, Se-HCH), 1.45 (d, *J* = 0.5 Hz, 3H, C-CH_3_), 1.37 (d, *J* = 0.5 Hz, C-CH_3_). ^13^C-NMR (CDCl_3_, 75 MHz); δ (ppm): 131.5, 128.3 (3C), 123.2, 109.7, 99.4, 75.3, 69.3, 68.9, 31.8, 27.0, 25.5. MS: *m/z* (rel. int.) 296 (M^+^, 8.7), 181 (43.8), 115 (25.9), 102 (26.4), 43 (100.0).

*2,2-Dimethyl-1,3-dioxolanylmethyl(hex-1-yn-1-yl)selane* **3b** (Table 1, entry 2) [41]: Yield: 0.087 g (63%); yellow oil. MS: *m/z* (rel. int.) 276 (M^+^, 8.5), 101 (39.1), 79 (22.4), 57 (31.0), 43 (100.0).

*2,2-Dimethyl-1,3-dioxolanylmethyl(4-methylphenylethynyl)selane* **3c** (Table 1, entry 3) [41]: Yield: 0.101 g (65%); yellow solid. m.p. 45–47 °C. MS: *m/z* (rel. int.) 310 (M^+^, 23.7), 195 (66.0), 115 (87.0), 57 (66.0), 43 (100.0).

*2,2-Dimethyl-1,3-dioxolanylmethyl(2-methylphenylethynyl)selane* **3d** (Table 1, entry 4) [41]: Yield: 0.109 g (70%); yellow oil. MS: *m/z* (rel. int.) 310 (M^+^, 9.3), 195 (12.4), 115 (100.0), 101 (10.0), 43 (39.7).

*2,2-Dimethyl-1,3-dioxolanylmethyl(4-methoxyphenylethynyl)selane* **3e** (Table 1, entry 5) [41]: Yield: 0.098 g (60%); yellow solid; m.p. 39–41 °C. ^1^H-NMR (CDCl_3_, 400 MHz); δ (ppm): 7.27 (d, *J* = 8.9 Hz, 2H, Ar-H), 6.74 (d, *J* = 8.9 Hz, 2H, Ar-H), 4.37–4.43 (m, 1H, O-CH), 4.12 (dd, *J* = 8.5 and 6.0 Hz, 1H, O-HCH), 3.79 (dd, *J* = 8.5 and 5.9 Hz, 1H, O-HCH), 3.71 (s, 3H, Ar-OCH_3_), 2.98 (dd, *J* = 12.1 and 5.1 Hz, 1H, Se-HCH), 2.84 (dd, *J* = 12.1 and 7.7 Hz, 1H, Se-HCH), 1.37 (s, 3H, C-CH_3_), 1.29 (s, 3H, C-CH_3_). ^13^C-NMR (CDCl_3_, 100 MHz); δ (ppm): 159.8, 133.3, 115.5, 113.9, 109.7, 99.3, 75.4, 69.0, 67.3, 55.2, 31.8, 27.0, 25.5. MS: *m/z* (rel. int.) 326 (M^+^, 3.4), 211 (39.3), 196 (20.2), 132 (57.1), 43 (100.0).

*2,2-Dimethyl-1,3-dioxolanylmethyl(2-methoxyphenylethynyl)selane* **3f** (Table 1, entry 6): Yield: 0.098 g (60%); yellow oil. ^1^H-NMR (CDCl_3_, 400 MHz); δ (ppm): 7.35–7.37 (m, 1H, Ar-H), 7.24–7.28 (m, 1H, Ar-H), 6.84–6.90 (m, 2H, Ar-H), 4.50–4.56 (m, 1H, O-CH), 4.21–4.25 (m, 1H, O-HCH), 3.93 (dd, *J* = 7.7 and 6.1 Hz, 1H, O-HCH), 3.86 (s, 3H, Ar-OCH_3_), 3.08 (dd, *J* = 12.0 and 4.0 Hz, 1H, Se-HCH), 2.93 (dd, *J* = 12.0 and 8.0 Hz, 1H, Se-HCH), 1.45 (s, 3H, C-CH_3_), 1.37 (s, 3H, C-CH_3_). ^13^C-NMR (CDCl_3_, 100 MHz); δ (ppm): 160.1, 133.3, 129.6, 120.3, 112.4, 110.5, 109.5, 95.7, 75.5, 73.0, 69.0, 55.6, 31.7, 26.9, 25.5. MS: *m/z* (rel. int.) 326 (M^+^, 29.4), 131 (100.0), 119 (51.2), 57 (71.9), 43 (91.4). HRMS: Calculated mass to C_15_H_18_O_3_Se: [M]^+^ 326.0421, found: 326.0439.

*2,2-Dimethyl-1,3-dioxolanylmethyl(4-fluorophenylethynyl)selane* **3g** (Table 1, entry 7) [41]: Yield: 0.105 g (67%); yellow solid; m.p. 37–39 °C. MS: *m/z* (rel. int.) 314 (M^+^, 0.3), 199 (32.2), 120 (27.1), 107 (90.6), 43 (100.0).

*2,2-Dimethyl-1,3-dioxolanylmethyl(cyclohex-1-en-1-ylethynyl)selane* **3h** (Table 1, entry 8): Yield: 0.078 g (52%); yellow oil. ^1^H-NMR (CDCl_3_, 400 MHz); δ (ppm): 6.07–6.09 (m, 1H, C=CH), 4.41–4.45 (m, 1H, O-CH), 4.18 (dd, *J* = 8.5 and 6.0 Hz, 1H, O-HCH), 3.83 (dd, *J* = 8.5 and 5.9 Hz, 1H, O-HCH), 2.99 (dd, *J* = 12.1 and 5.0 Hz, 1H, Se-HCH), 2.84 (dd, *J* = 12.1 and 8.0 Hz, 1H, Se-HCH), 2.09–2.10 (m, 4H), 1.55–1.65 (m, 4H), 1.43 (s, 3H, C-CH_3_), 1.36 (s, 3H, C-CH_3_). ^13^C-NMR (CDCl_3_, 100 MHz); δ (ppm): 135.3, 121.0, 109.6, 101.5, 75.5, 69.0, 65.7, 31.6, 29.1, 27.0, 25.61, 25.56, 22.3, 21.4. MS: *m/z* (rel. int.) 300 (M^+^, 6.0), 185 (6.7), 104 (35.1), 91 (19.9), 43 (100.0). HRMS: Calculated mass to C_14_H_20_O_2_Se: [M + H]^+^ 301.0707, found: 301.0690.

### 3.3. General Procedure for the Synthesis of Organotellanyl Alkynes ***3i***–***m***

To a solution of alkyne **1** (1.0 mmol) in THF (5.0 mL) under N_2_ atmosphere, BuLi (1.6 mol/L in hexanes; 1.0 mmol) was added at 0 °C. After 20 min, the temperature was increased to room temperature and elemental tellurium (Te^0^, 1.0 mmol) was added. The stirring at room temperature was maintained until all tellurium has been consumed and then a solution of racemic tosyl solketal **2** (0.5 mmol) in THF (2.0 mL) was added and the mixture was stirred under reflux for additional 1.5 h. After, the reaction mixture was quenched with water (15.0 mL) and extracted with ethyl acetate (3 × 15.0 mL). The organic phase was separated, dried over MgSO_4_, and the solvent was evaporated under reduced pressure. The product was isolated by column chromatography using hexanes/ethyl acetate as eluent.

#### Analytical Data of Products **3i**–**m**

*2,2-Dimethyl-1,3-dioxolanylmethyl(phenylethynyl)tellane* **3i** (Table 1, entry 9) [41]: Yield: 0.147 g (85%); red oil. ^1^H-NMR (CDCl_3_, 400 MHz); δ (ppm): 7.31–7.34 (m, 2H, Ar-H), 7.18–7.25 (m, 3H, Ar-H), 4.38–4.44 (m, 1H, O-CH), 4.12 (dd, *J* = 8.4 and 6.1 Hz, 1H, O-HCH), 3.73 (dd, *J* = 8.4 and 6.1 Hz, 1H, O-HCH), 3.00–3.08 (m, 2H, Te-CH_2_), 1.37 (s, 3H, C-CH_3_), 1.28 (s, 3H, C-CH_3_). ^13^C-NMR (CDCl_3_, 100 MHz); δ (ppm): 131.7, 128.3, 128.2, 123.4, 111.3, 109.7, 75.9, 70.0, 44.2, 27.0, 25.6, 13.6. MS: *m/z* (rel. int.) 346 (M^+^, 32.7), 231 (58.2), 101 (75.7), 57 (100.0), 43 (77.0).

*2,2-Dimethyl-1,3-dioxolanylmethyl(hex-1-yn-1-yl)tellane* **3j** (Table 1, entry 10): Yield: 0.103 g (63%); red oil. ^1^H-NMR (CDCl_3_, 500 MHz); δ (ppm): 4.41–4.46 (m, 1H, O-CH), 4.18 (dd, *J* = 8.4 and 6.0 Hz, 1H, O-HCH), 3.77 (dd, *J* = 8.4 and 6.4 Hz, 1H, O-HCH), 3.02 (dd, *J* = 11.7 and 5.2 Hz, 1H, Te-HCH), 2.97 (dd, *J* = 11.7 and 7.6 Hz, 1H, Te-HCH), 2.48 (t, *J* = 7.0 Hz, 2H, CH_2_C_sp_), 1.47–1.53 (m, 2H), 1.43 (s, 3H, C-CH_3_), 1.37–1.43 (m, 2H), 1.35 (s, 3H, C-CH_3_), 0.91 (t, *J* = 7.3 Hz, 3H). ^13^C-NMR (CDCl_3_, 125 MHz); δ (ppm): 112.8, 109.7, 76.2, 70.0, 31.0, 27.0, 25.6, 21.9, 20.6, 13.5, 12.4. MS: *m/z* (rel. int.) 326 (M^+^, 21.5), 115 (63.0), 81 (79.9), 57 (100.0), 43 (77.2). HRMS: Calculated mass for C_12_H_20_O_2_Te: [M + OH]^+^ 343.0553, found: 343.0533.

*2,2-Dimethyl-1,3-dioxolanylmethyl(4-methylphenylethynyl)tellane* **3k** (Table 1, entry 11): Yield: 0.099 g (55%); red oil. ^1^H-NMR (CDCl_3_, 500 MHz); δ (ppm): 7.29 (d, *J* = 8.1 Hz, 2H, Ar-H), 7.10 (d, *J* = 8.1 Hz, 2H, Ar-H), 4.45–4.50 (m, 1H, O-CH), 4.20 (dd, *J* = 8.4 and 6.0 Hz, 1H, O-HCH), 3.81 (dd, *J* = 8.4 and 6.4 Hz, 1H, O-HCH), 3.10–3.11 (m, 2H, Te-CH_2_), 2.35 (s, 3H, Ar-CH_3_), 1.45 (s, 3H, C-CH_3_), 1.35 (s, 3H, C-CH_3_). ^13^C-NMR (CDCl_3_, 125 MHz); δ (ppm): 138.6, 131.7, 128.9, 120.4, 111.4, 109.7, 76.0, 70.0, 42.9, 27.0, 25.6, 21.4, 13.5. MS: *m/z* (rel. int.) 360 (M^+^, 12.8), 245 (27.8), 115 (100.0), 57 (78.9), 43 (75.2). HRMS: Calculated mass for C_15_H_18_O_2_Te: [M + H]^+^ 361.0447, found: 361.0469.

*2,2-Dimethyl-1,3-dioxolanylmethyl(cyclohex-1-en-1-ylethynyl)tellane* **3l** (Table 1, entry 12): Yield: 0.107 g (61%); yellow oil; ^1^H-NMR (CDCl_3_, 500 MHz); δ (ppm): 6.06–6.09 (m, 1H, C=CH), 4.42–4.47 (m, 1H, O-CH), 4.19 (dd, *J* = 8.4 and 5.9 Hz, 1H, O-HCH ), 3.78 (dd, *J* = 8.4 and 6.4 Hz, 1H, O-HCH), 3.02–3.03 (m, 2H, Te-CH_2_), 2.08–2.16 (m, 4H), 1.54–1.65 (m, 4H), 1.44 (s, 3H, C-CH_3_), 1.35 (s, 3H, C-CH_3_). ^13^C-NMR (CDCl_3_, 125 MHz); δ (ppm): 135.9, 121.1, 113.5, 109.7, 76.2, 70.0, 39.9, 29.2, 27.0, 25.6, 25.5, 22.2, 21.4, 13.2. MS: *m/z* (rel. int.) 350 (M^+^, 40.3), 115 (31.8), 105 (100.0), 57 (92.9), 43 (73.8). HRMS: Calculated mass for C_14_H_20_O_2_Te: [M + H]^+^ 351.0604, found: 351.0597.

*2,2-Dimethyl-1,3-dioxolanylmethyl(4-tert-buthylphenylethynyl)tellane* **3m** (Table 1, entry 13): Yield: 0.127 g (63%); red oil. ^1^H NMR (CDCl_3_, 500 MHz); δ (ppm): 7.31–7.35 (m, 4H, Ar-H), 4.46–4.51 (m, 1H, O-CH), 4.21 (dd, *J* = 8.4 and 6.0 Hz, 1H, O-HCH ), 3.81 (dd, *J* = 8.4 and 6.4 Hz, 1H, O-HCH), 3.10–3.11 (m, 2H, Te-CH_2_), 1.45 (s, 3H, C-CH_3_), 1.35 (s, 3H, C-CH_3_), 1.30 (s, 9H, C-CH_3_). ^13^C NMR (CDCl_3_, 125 MHz); δ (ppm): 151.7, 131.6, 125.2, 120.4, 111.5, 109.7, 76.1, 70.0, 42.9, 34.7, 31.1, 27.0, 25.6, 13.5. MS: *m/z* (rel. int.) 402 (M^+^, 26.2), 287 (19.6), 143 (66.4), 57 (99.7), 43 (100.0). HRMS: Calculated mass for C_18_H_24_O_2_Te: [M + H]^+^ 403.0917, found: 403.0908.

### 3.4. General Procedure for the Synthesis of 3-(Organotellanyl)propane-1,2-diol ***4a***–***c***

To a solution of the respective organotellanyl alkyne **3** (1.0 mmol) in MeOH (2.5 mL) Dowex^®^ acidic ion-exchange resin (50WX8 20–50 mesh; 1.122 g) was added at room temperature. The reaction mixture was stirred for 5 h at room temperature and then it was filtered and washed with MeOH. The filtrate was concentrated and chromatographed (50% EtOAc/hexanes).

#### Analytical Data of Products **4a**–**c**

*3-(Phenylethynyltellanyl)propane-1,2-diol* **4a** (Table 2, entry 1): Yield: 0.153 g (50%); red oil. ^1^H-NMR (CDCl_3_, 400 MHz); δ (ppm): 7.39–7.41 (m, 2H, Ar-H), 7.26–7.32 (m, 3H, Ar-H), 4.04–4.09 (m, 1H, O-CH), 3.81 (dd, *J* = 11.3 and 3.1 Hz, 1H, O-HCH), 3.66 (dd, *J* = 11.3 and 6.1 Hz, 1H, O-HCH), 2.81–3.07 (m, 4H, Te-CH_2_ and 2 O-H). ^13^C-NMR (CDCl_3_, 100 MHz); δ (ppm): 131.8, 128.4, 128.2, 123.4, 111.2, 71.5, 66.6, 45.1, 14.8. MS: *m/z* (rel. int.) 306 (M^+^, 7.9), 231 (11.7), 155 (44.9), 102 (43.7), 91 (100.0). HRMS: Calculated mass for C_11_H_12_O_2_Te: [M + H]^+^ 306.9978, found: 306.9903.

*3-(Cyclohex-1-en-1-ylethynyltellanyl)propane-1,2-diol* **4b** (Table 2, entry 2): Yield: 0.155 g (50%); yellow oil. ^1^H-NMR (CDCl_3_, 400 MHz); δ (ppm): 6.07–6.10 (m, 1H, C=CH), 4.0–4.06 (m, 1H, O-CH), 3.79 (dd, *J* = 11.3 and 3.4 Hz, 1H, O-HCH ), 3.65 (dd, *J* = 11.3 and 6.1 Hz, 1H, O-HCH), 2.76–3.0 (m, 4H, Te-CH_2_ and 2 O-H), 2.07–2.17 (m, 4H), 1.54–1.65 (m, 4H). ^13^C-NMR (CDCl_3_,100 MHz); δ (ppm): 136.1, 121.1, 113.5, 71.7, 66.5, 40.7, 29.3, 25.5, 22.2, 21.4, 14.5. MS: *m/z* (rel. int.) 310 (M^+^, 20.6), 235 (6.8), 105 (100.0), 91 (43.0), 79 (54.0). HRMS: Calculated mass for C_11_H_16_O_2_Te: [M + H]^+^ 311.0291, found: 311.0273.

*3-(4-tert-Buthylphenylethynyltellanyl)propane-1,2-diol* **4c** (Table 2, entry 3): Yield: 0.170 g (47%); red oil. ^1^H-NMR (CDCl_3_, 400 MHz); δ (ppm): 7.29–7.35 (m, 4H, Ar-H), 4.03–4.09 (m, 1H, O-CH), 3.80 (dd, *J* = 11.4 and 3.1 Hz, 1H, O-HCH), 3.64 (dd, *J* = 11.4 and 6.2 Hz, 1H, O-HCH), 3.03–3.30 (m, 4H, Te-CH_2_ and 2 O-H), 1.28 (s, 9H, C-CH_3_). ^13^C-NMR (CDCl_3_, 100 MHz); δ (ppm): 151.8, 131.6, 125.2, 120.4, 111.3, 71.6, 66.5, 44.3, 34.7, 31.1, 14.7. MS: *m/z* (rel. int.) 362 (M^+^, 20.2), 288 (11.4), 143 (100), 57 (98.2), 41 (44.1). HRMS: Calculated mass for C_15_H_20_O_2_Te: [M + H]^+^ 363.0604, found: 363.0583.

### 3.5. Procedure for the Synthesis of the 3-Iodo-2-(2,2-dimethyl-1,3-dioxolanylmethyl)selenanylbenzo[b]furan ***5***

To a solution of *2,2-dimethyl-1,3-dioxolanylmethyl(2-methoxyphenylethynyl)selane* **3f** (0.25 mmol) in CH_2_Cl_2_ (2.0 mL) a solution of I_2_ (0.28 mmol) in CH_2_Cl_2_ (3.0 mL) was added. The reaction mixture was stirred for 1 h at room temperature. Then, saturated aqueous Na_2_S_2_O_3_ was added to remove the excess of I_2_. The mixture was then extracted with ethyl acetate (3 × 10 mL) and the organic phase was separated, dried over MgSO_4_ and concentrated under vacuum. The product was isolated by column chromatography using hexanes/ethyl acetate as eluent.

#### Analytical Data of Product **5**

*3-Iodo-2-(2,2-dimethyl-1,3-dioxolanylmethyl)selenanylbenzo[b]furan* **5** (Scheme 2): Yield: 0.093 g (85%); yellow oil; ^1^H-NMR (CDCl_3_, 400 MHz); δ (ppm): 7.44–7.47 (m, 1H, Ar-H), 7.28–7.37 (m, 3H, Ar-H), 4.36–4.42 (m, 1H, O-CH), 4.18 (dd, *J* = 8.5 and 6.0 Hz, 1H, O-HCH), 3.79 (dd, *J* = 8.5 and 6.0 Hz, 1H, O-HCH), 3.31 (dd, *J* = 12.3 and 5.2 Hz, 1H, Se-HCH), 3.11 (dd, *J* = 12.3 and 7.8 Hz, 1H, Se-HCH), 1.45 (s, 3H, C-CH_3_), 1.36 (s, 3H, C-CH_3_). ^13^C-NMR (CDCl_3_, 100 MHz); δ (ppm): 156.7, 146.6, 131.3, 125.6, 123.6, 121.2, 111.0; 109.8; 75.8, 75.4; 69.1; 30.8, 27.0; 25.5. MS: *m/z* (rel. int.) 438 (M^+^, 23.4), 168 (18.2), 115 (35.3), 101 (21.5), 57 (100.0). HRMS: Calculated mass for C_14_H_15_IO_3_Se: [M + NH_4_]^+^ 455.9575, found: 455.9577.

## 4. Conclusions

In summary, we developed a new and general protocol to prepare glycerol-derived organoselanyl and organotellanyl alkynes using tosyl solketal. Eight organoselanyl and five organotellanyl alkynes were obtained in good yields and short reaction times, when compared to previously described procedures. Some of the organotellanyl alkynes were deprotected using Dowex 50WX8-(H^+^) to give new water-soluble 3-organotellanylpropane-1,2-diols.

## Figures and Tables

**Table 1:**
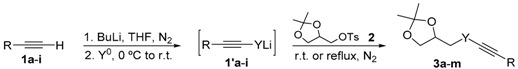
Scope of the synthesis of organoselanyl **3a**–**h** and organotellanyl alkynes **3i**–**m** ^a^.

Entry	Alkyne 1	Y	Product 3	Yield (%) ^b^
1	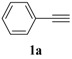	Se	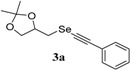	80
2	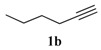	Se	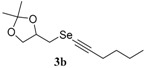	63
3	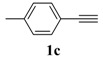	Se	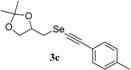	65
4	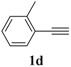	Se	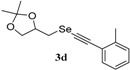	70
5	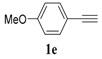	Se	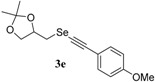	60
6	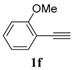	Se	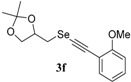	60
7	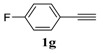	Se	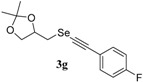	67
8	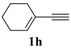	Se	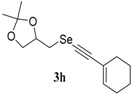	52
9	**1a**	Te	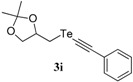	85
10	**1b**	Te	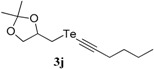	63
11	**1c**	Te	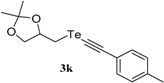	55
12	**1h**	Te	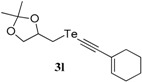	61
13	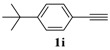	Te	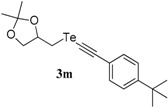	63

^a^ Reaction was performed using alkyne **1** (1.0 mmol), Se^0^ or Te^0^ (1.0 mmol), butyllithium solution (1.6 mol/L in hexanes; 1.0 mmol) at 0 °C in THF (5.0 mL) under N_2_ atmosphere. Then, tosyl solketal **2** (0.5 mmol) in THF (2.0 mL) was added at r.t. and the mixture stirred for additional 3 h (**3a**–**h**) or under reflux for 1.5 h (**3i**–**m**); ^b^ Yields are given for isolated products.

**Table 2:**
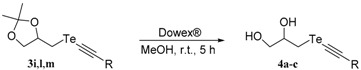
Synthesis of new 3-organotellanylpropane-1,2-diols **4a**–**c** ^a^.

Entry	Tellanyl Alkyne 3	Diol 4	Solubility (mg/mL) ^b^	Yield (%) ^c^
1	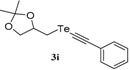	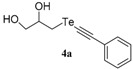	2.3	50
2	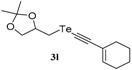	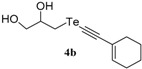	2.8	50
3	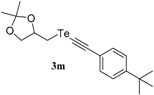	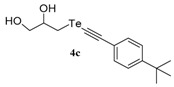	1.5	47

^a^ Reaction was performed using 1.0 mmol of **3**, 1.112 g of Dowex^®^ in 2.5 mL of MeOH at r.t. for 5 h; ^b^ Solubility measured in water; ^c^ Yields are given for isolated products.

## Supplementary Materials

Supplementary materials are available at http://www.mdpi.com/1420-3049/22/3/391/s1.
