# Supplementary material for: Glycerol as Precursor of Organoselanyl and Organotellanyl Alkynes
Source: Molecules. 2017 Mar 2;22(3):391. doi: 10.3390/molecules22030391 (PMC6155406; doi:10.3390/molecules22030391)
Supplement: Supplementary file 1 [file molecules-22-00391-s001.pdf]

Supporting Information

Glycerol as Precursor to Organoselanyl and Organotellanyl Alkynes

Eder J. Lenardão, Elton L. Borges, Guilherme Stach, Liane K. Soares, Diego Alves, Ricardo F. Schumacher, Luana Bagnoli, Francesca Marini and Gelson Perin

Selected Spectra

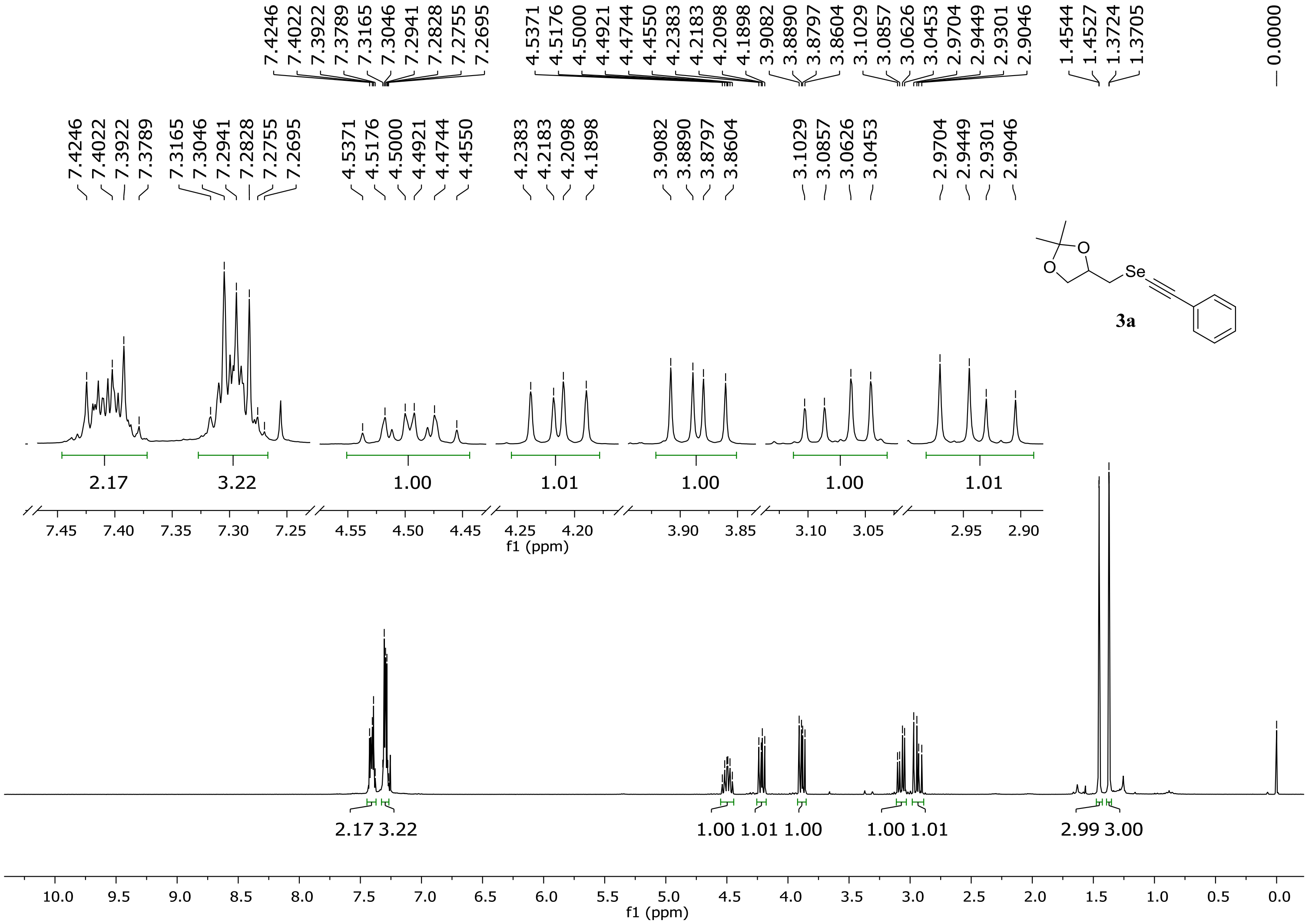

Figure S1. ^{1}H NMR ($CDCl_3$, 300 MHz) of the product **3a**.

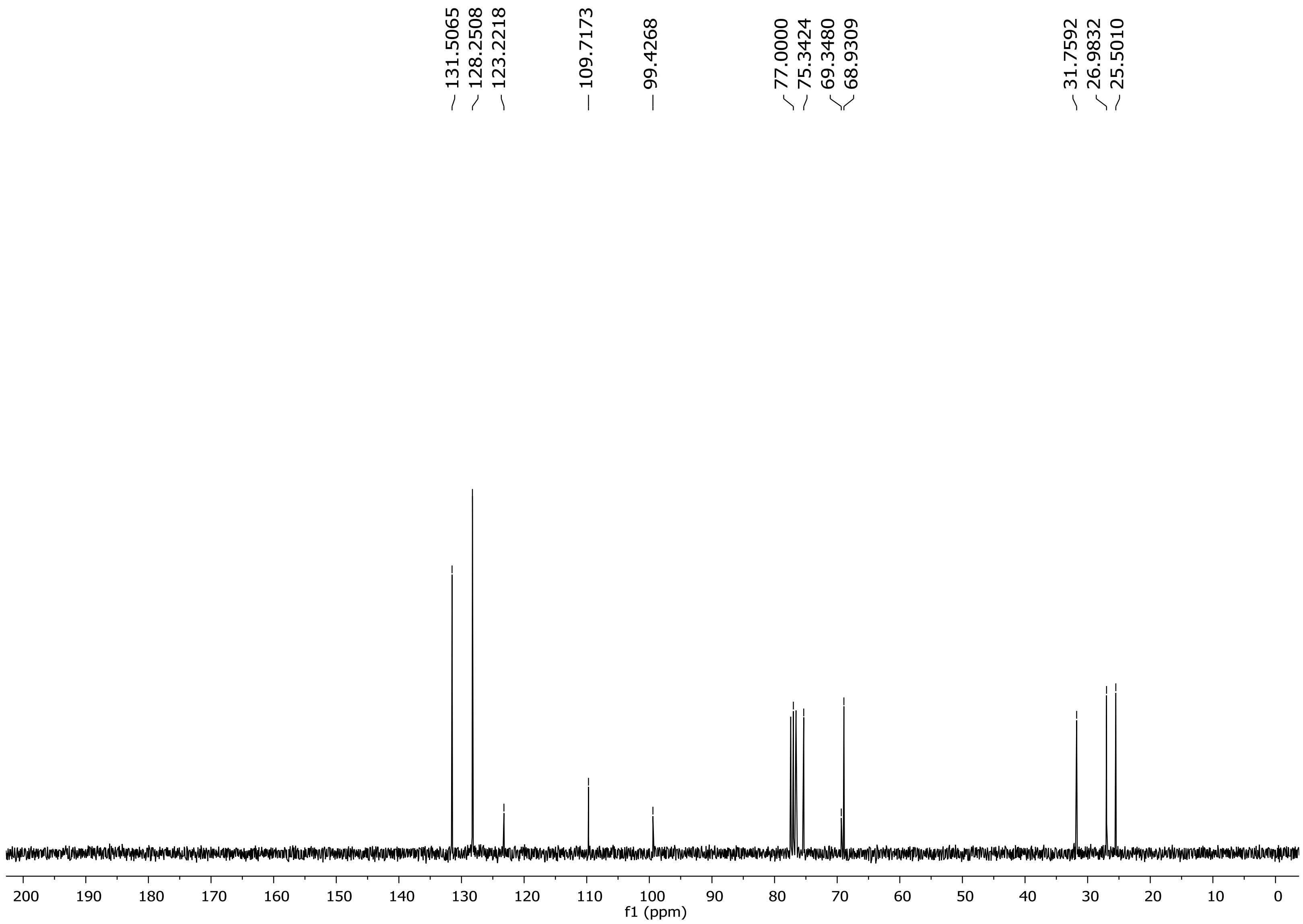

Figure S2. ^{13}C NMR ($CDCl_3$, 75 MHz) of the product **3a**.

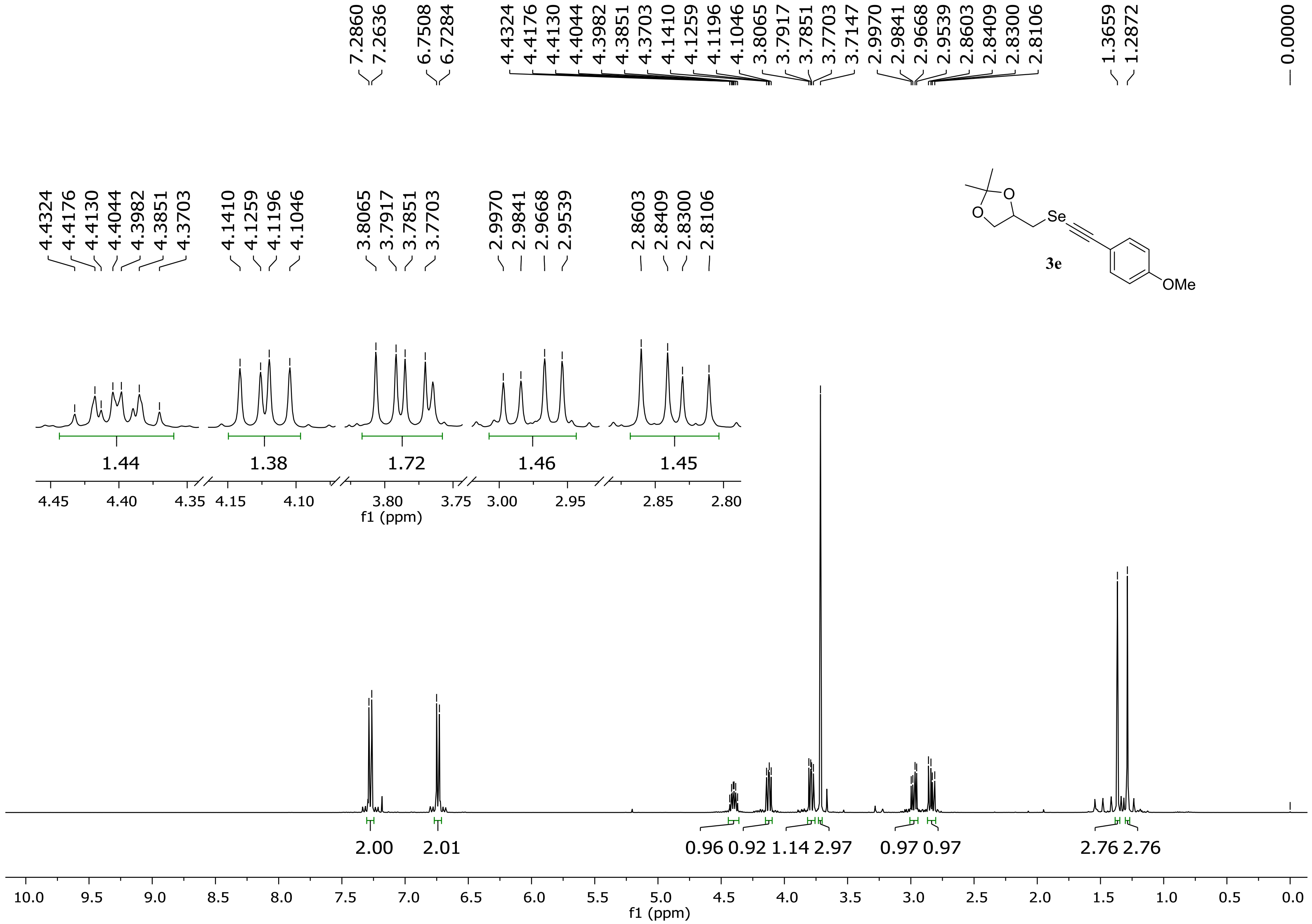

Figure S3. ^{1}H NMR ($CDCl_3$, 400 MHz) of the product **3e**.

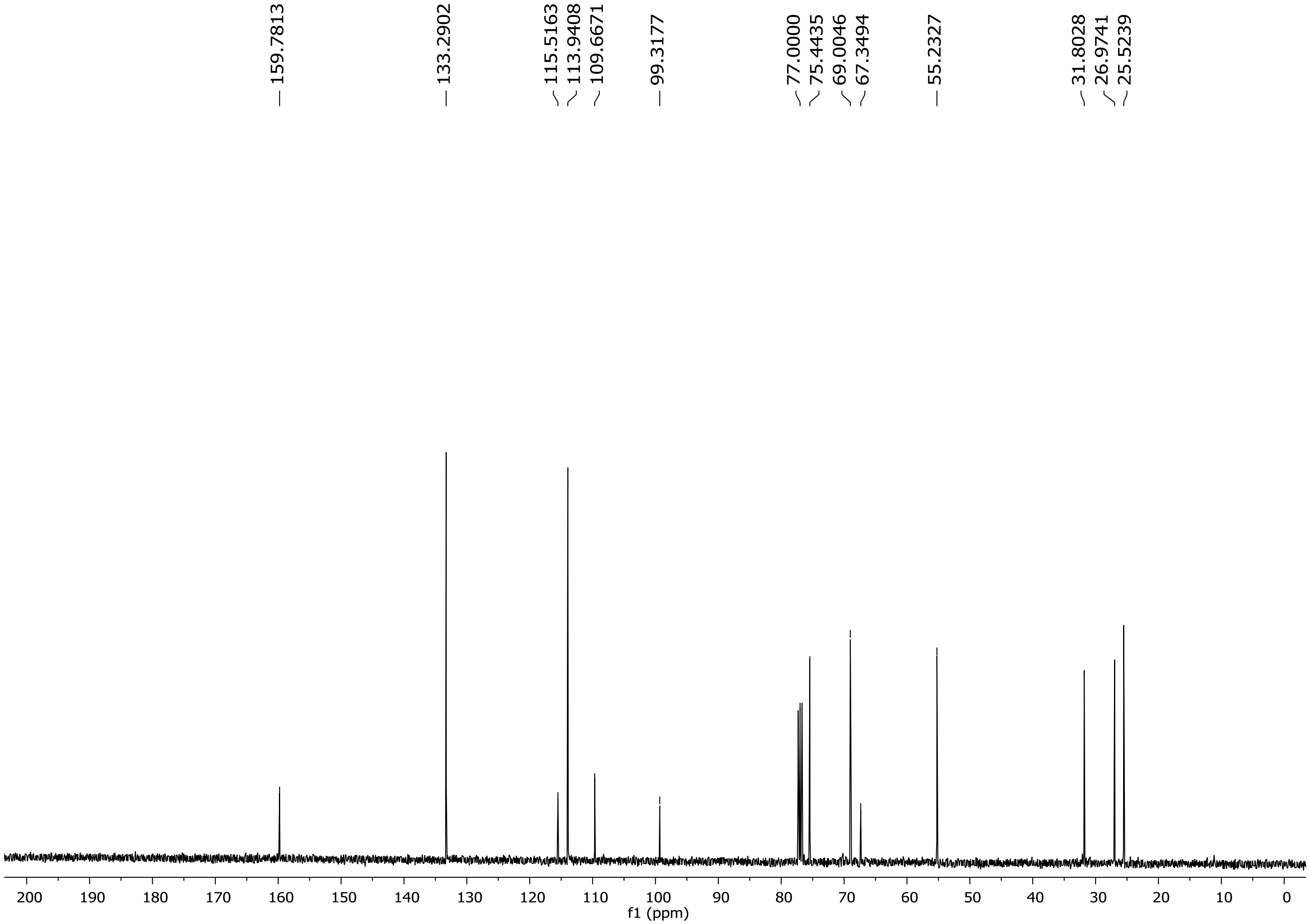

Figure S4. ^{13}C NMR ($CDCl_3$, 100 MHz) of the product **3e**.

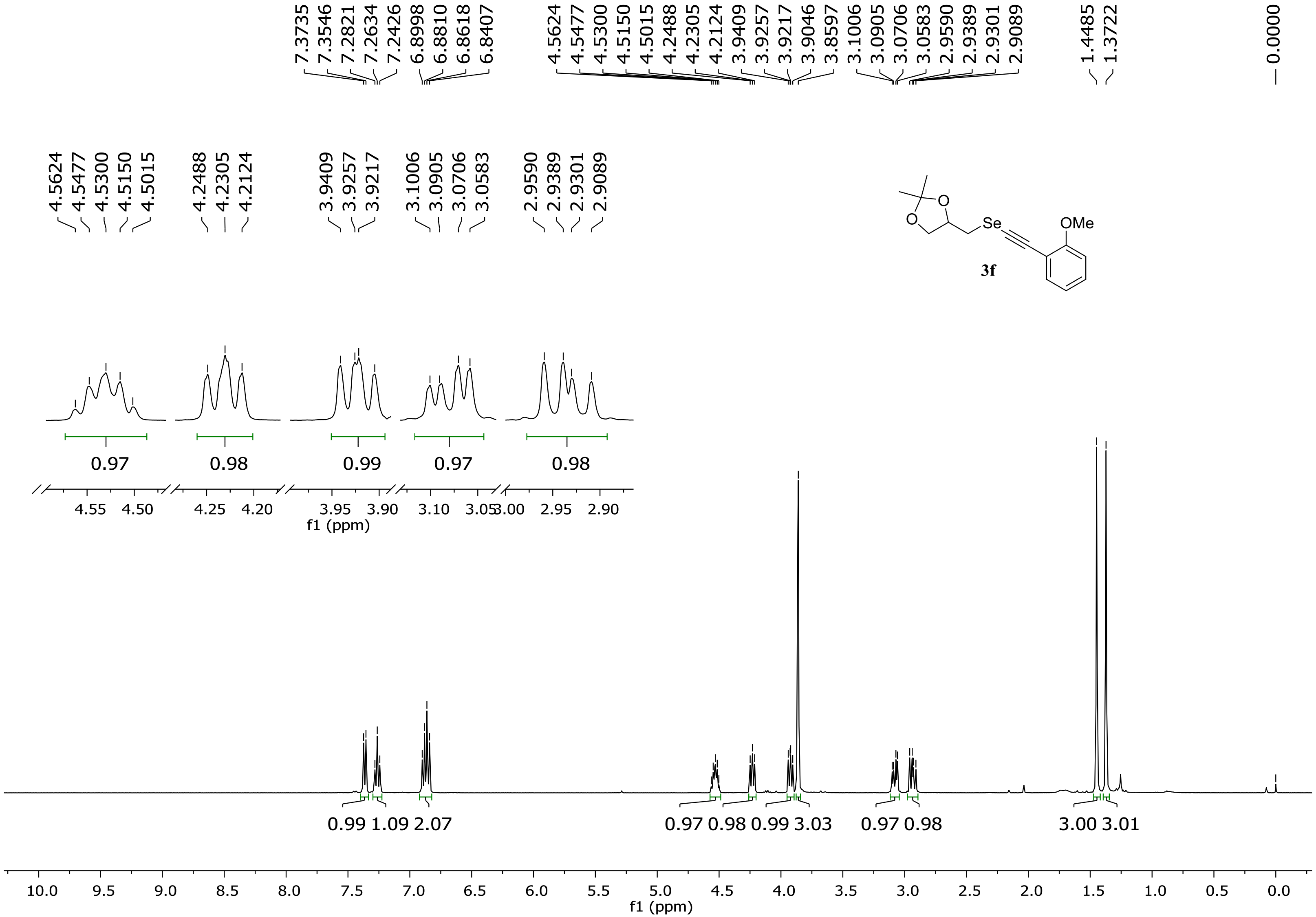

Figure S5. ^{1}H NMR ($CDCl_3$, 400 MHz) of the product **3f**.

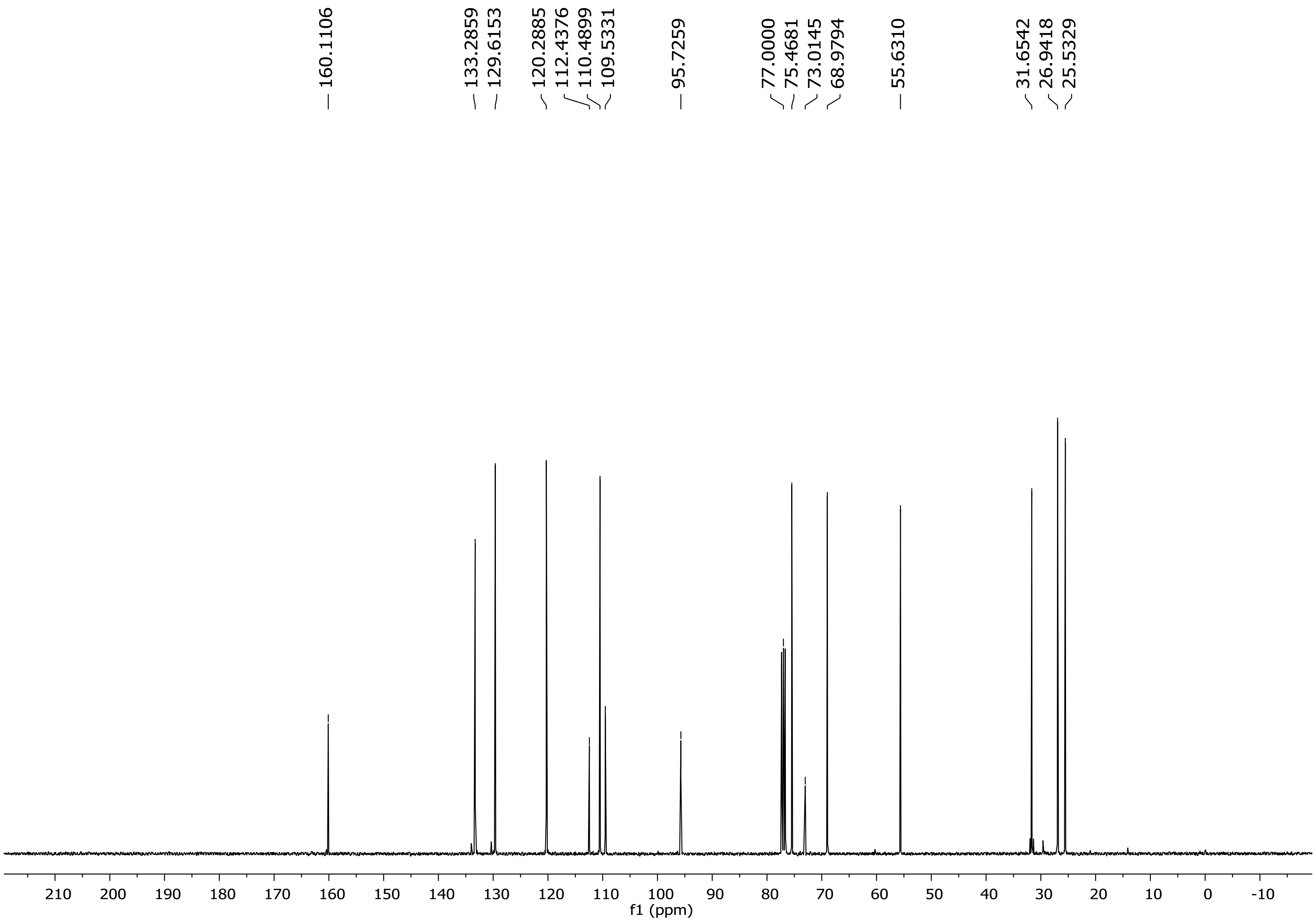

Figure S6. ^{13}C NMR ($CDCl_3$, 100 MHz) of the product **3f**.

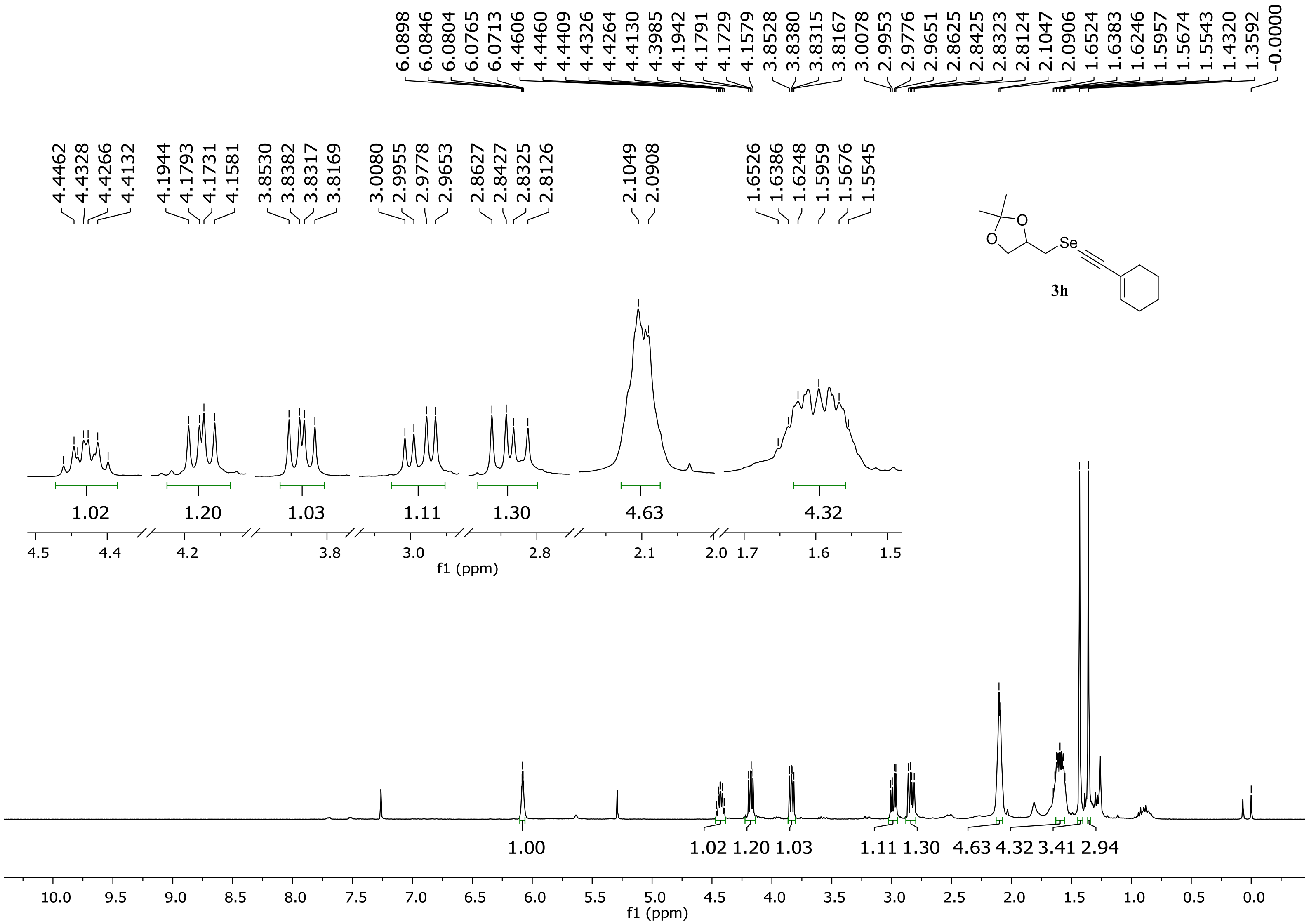

Figure S7. ^{1}H NMR ($CDCl_3$, 400 MHz) of the product **3h**.

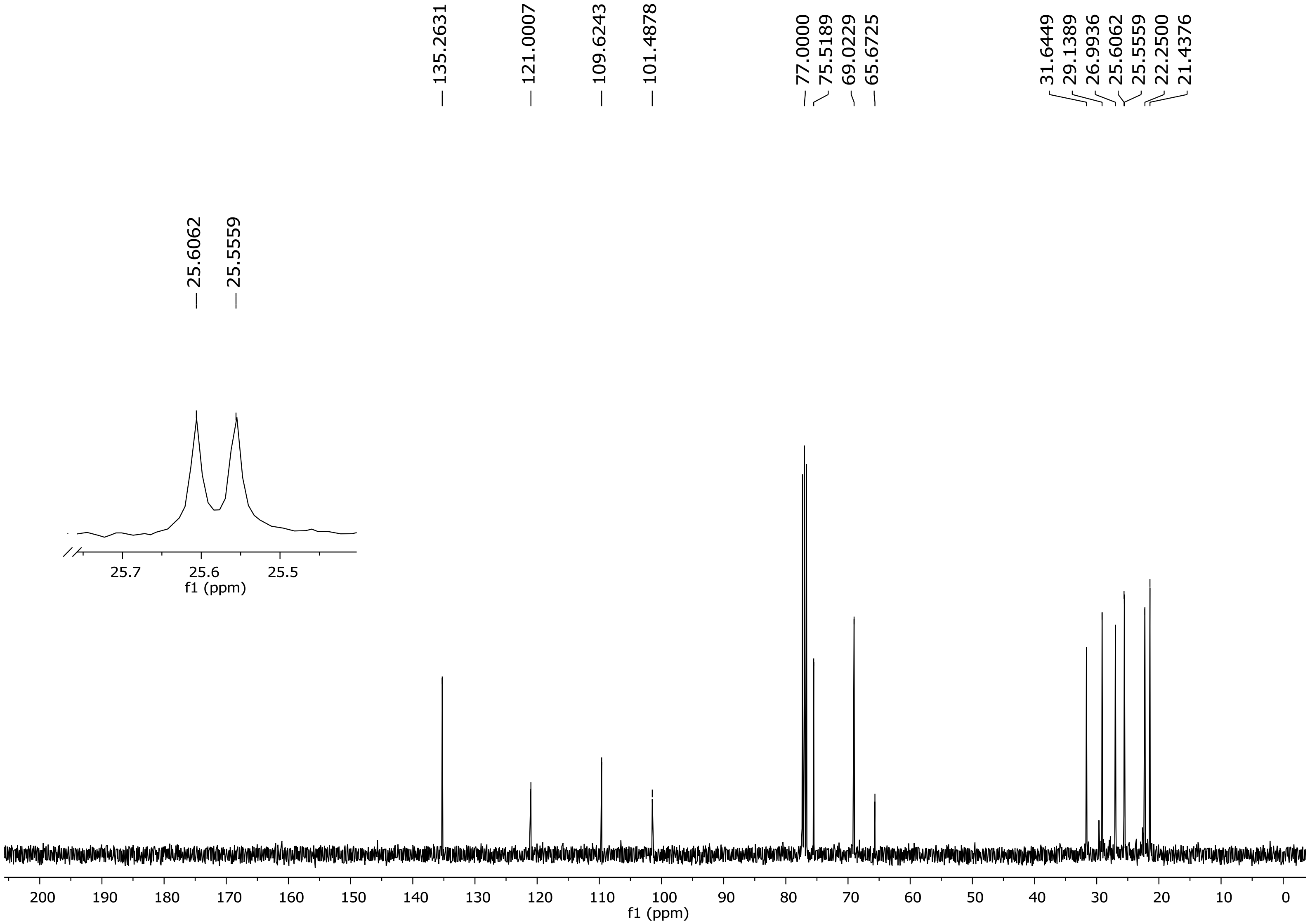

Figure S8. ^{13}C NMR ($CDCl_3$, 100 MHz) of the product **3h**.

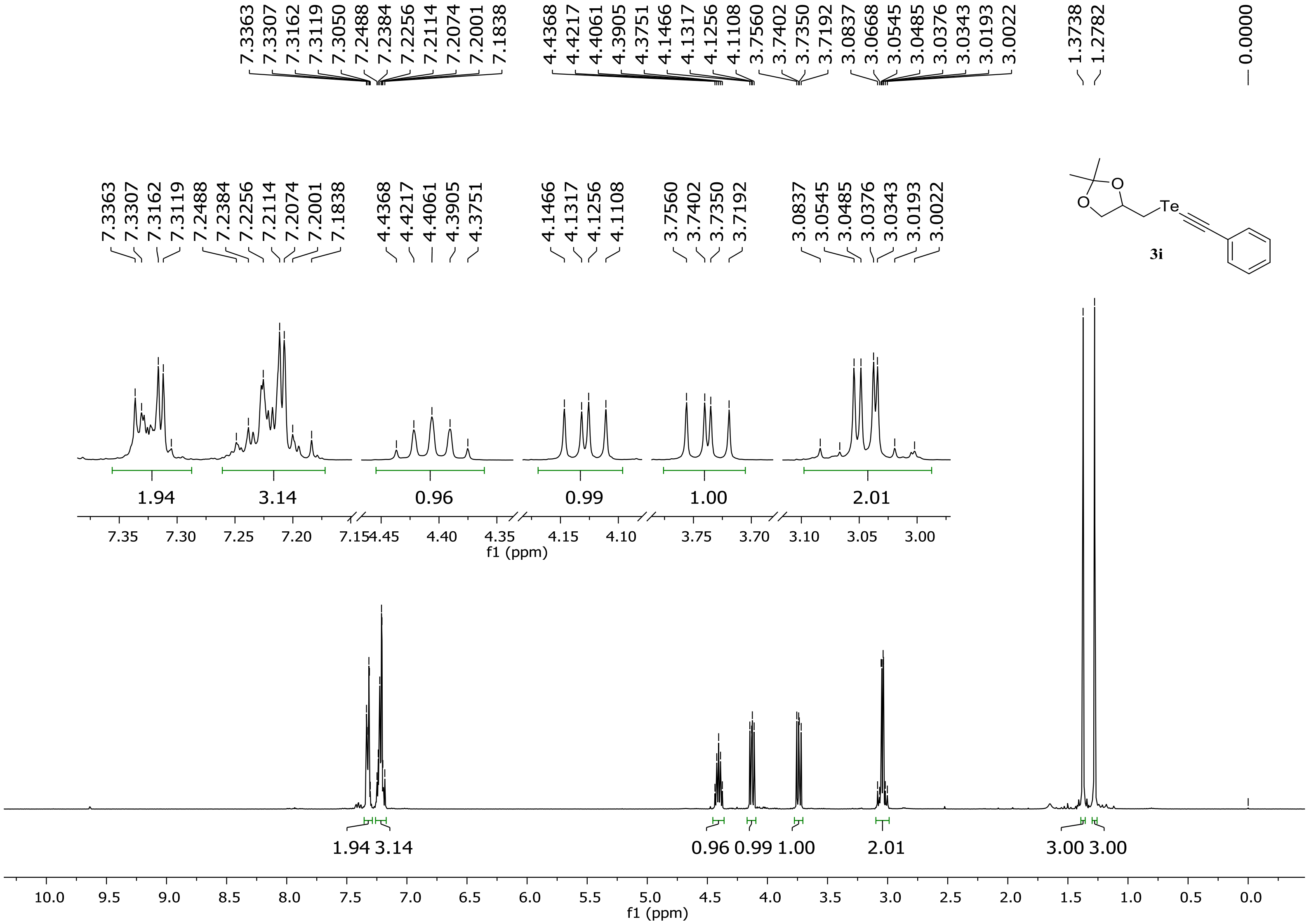

Figure S9. ^{1}H NMR ($CDCl_3$, 400 MHz) of the product **3i**.

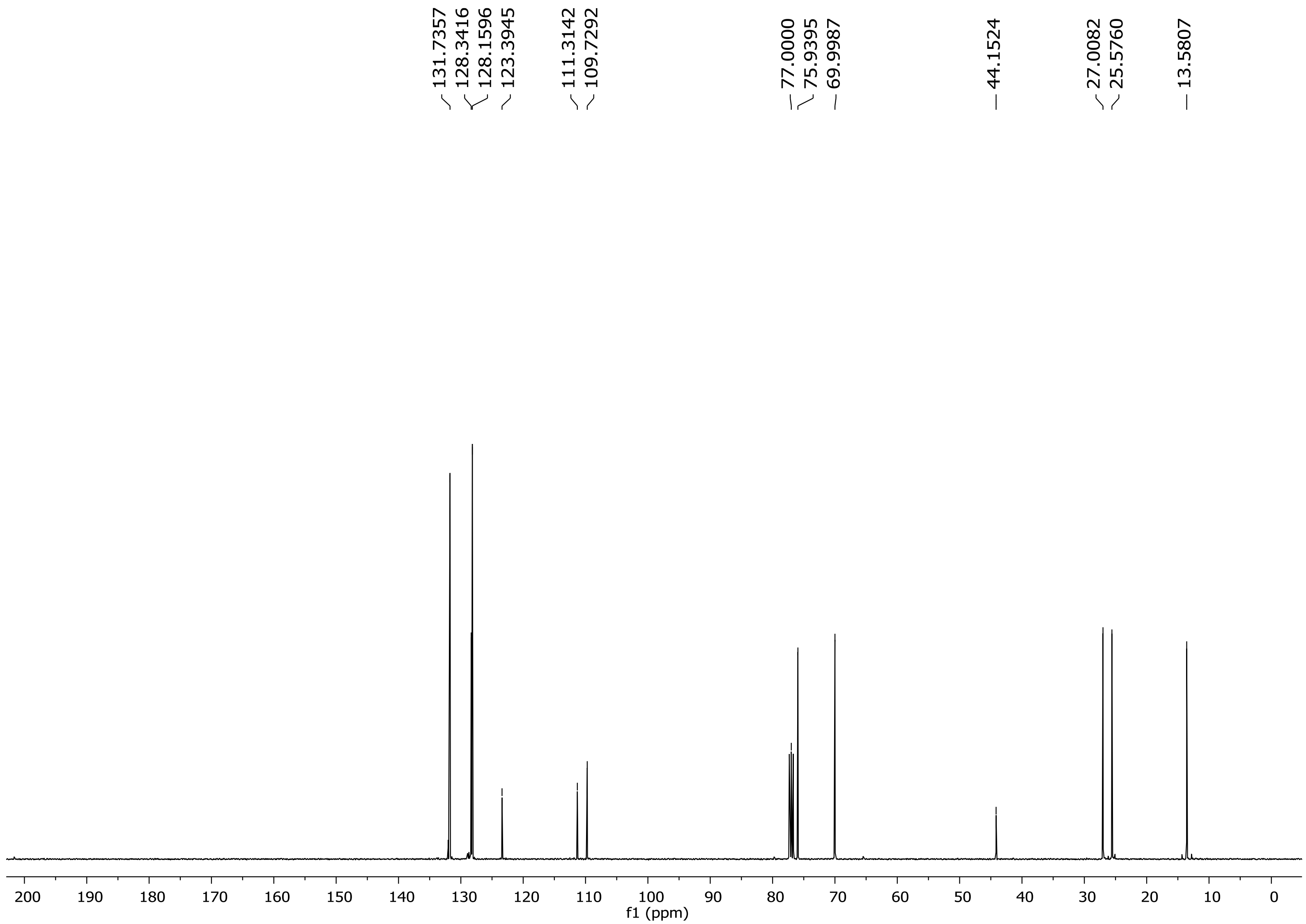

Figure S10. ^{13}C NMR ($CDCl_3$, 100 MHz) of the product **3i**.

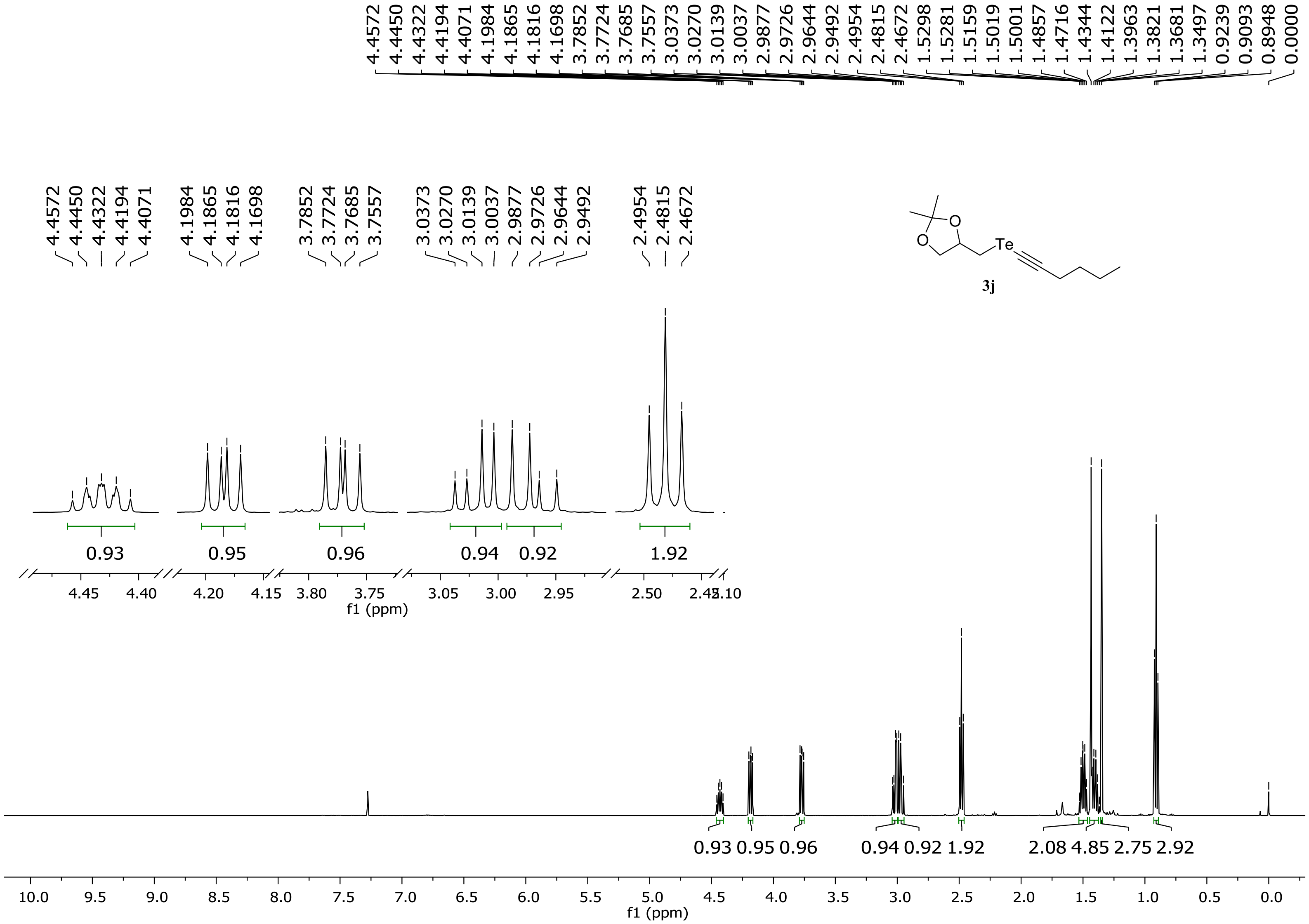

Figure S11. ^{1}H NMR ($CDCl_3$, 500 MHz) of the product **3j**.

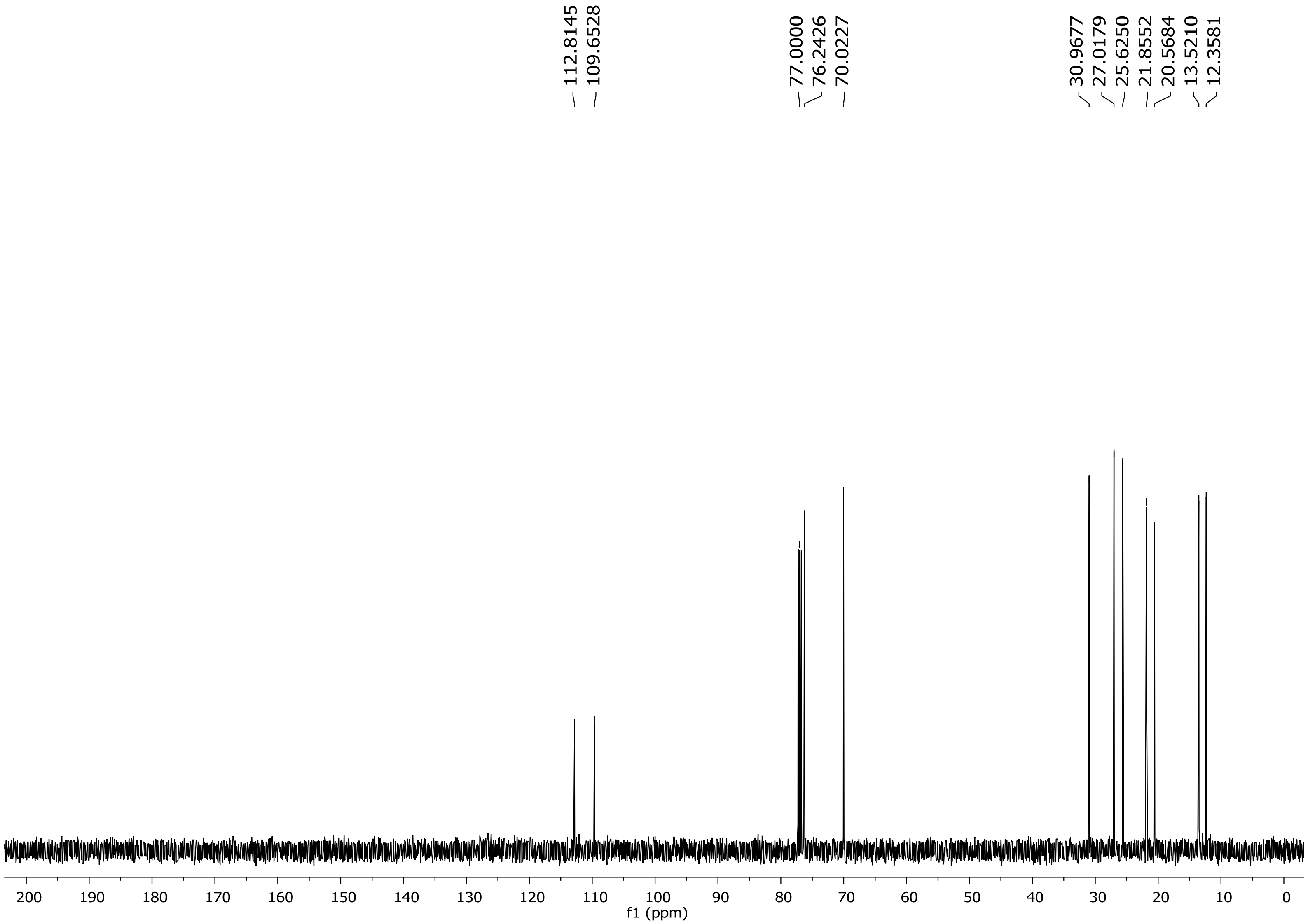

Figure S12. ^{13}C NMR ($CDCl_3$, 125 MHz) of the product **3j**.

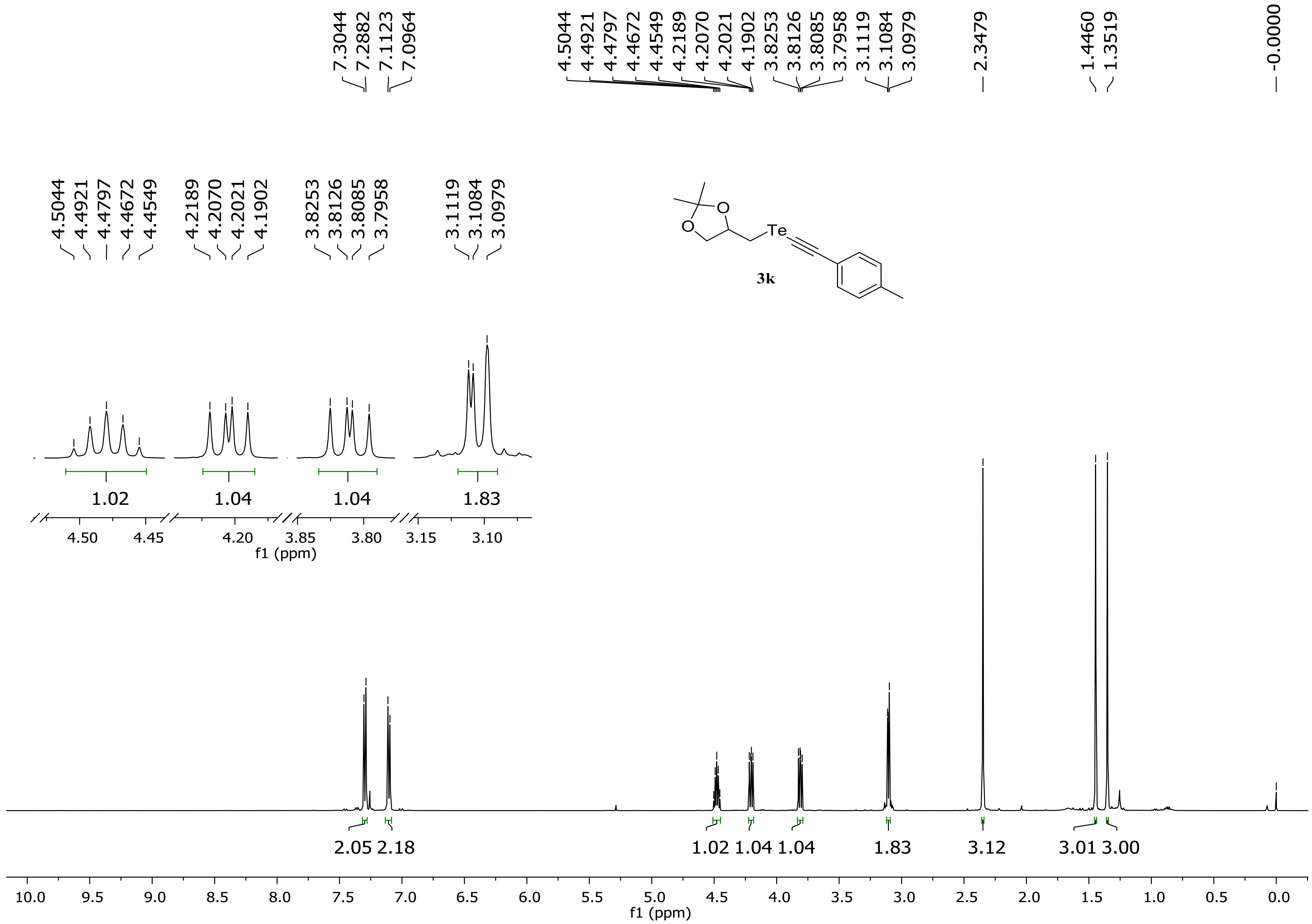

Figure S13. ^{1}H NMR ($CDCl_3$, 500 MHz) of the product **3k**.

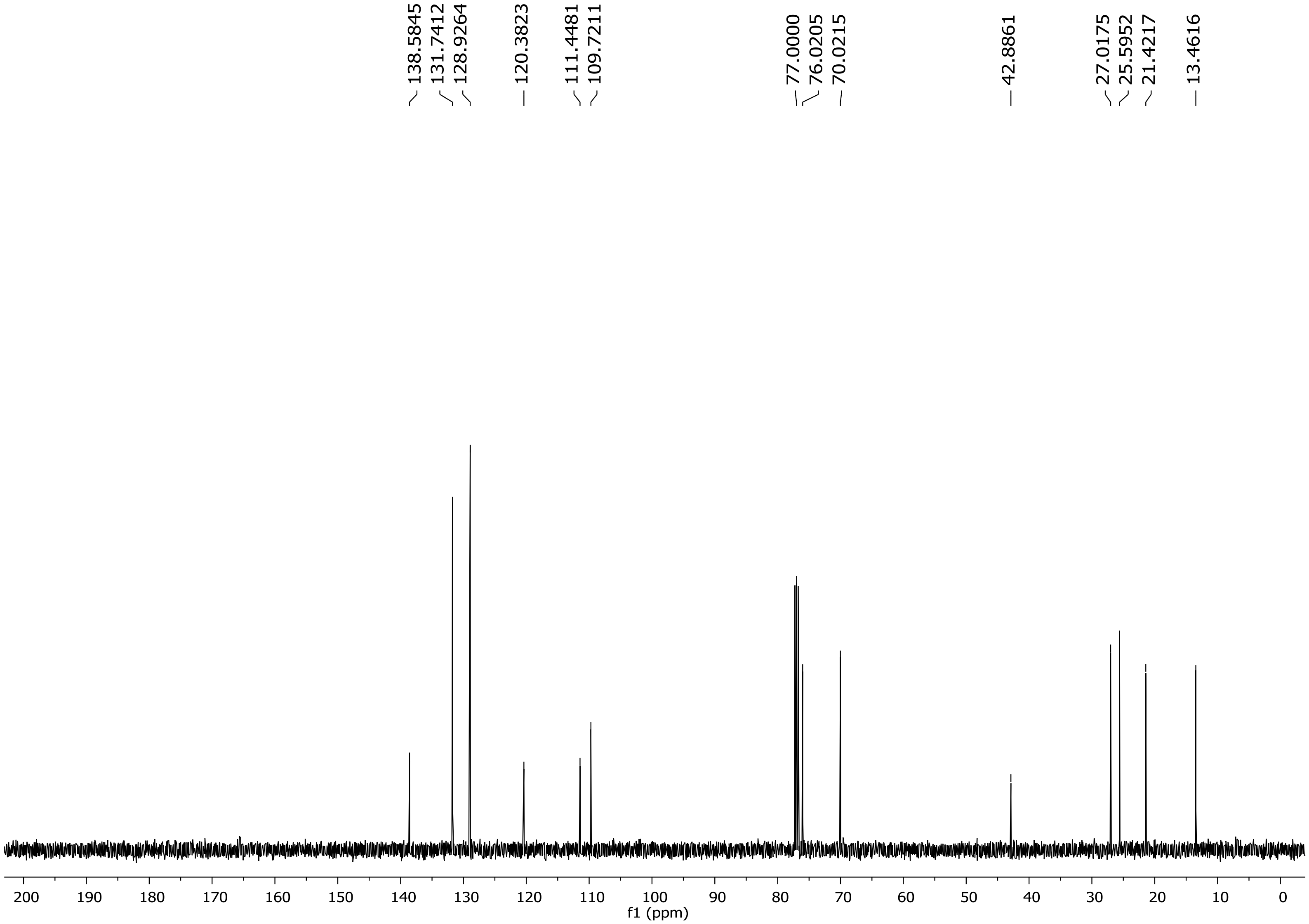

Figure S14. ^{13}C NMR ($CDCl_3$, 125 MHz) of the product **3k**.

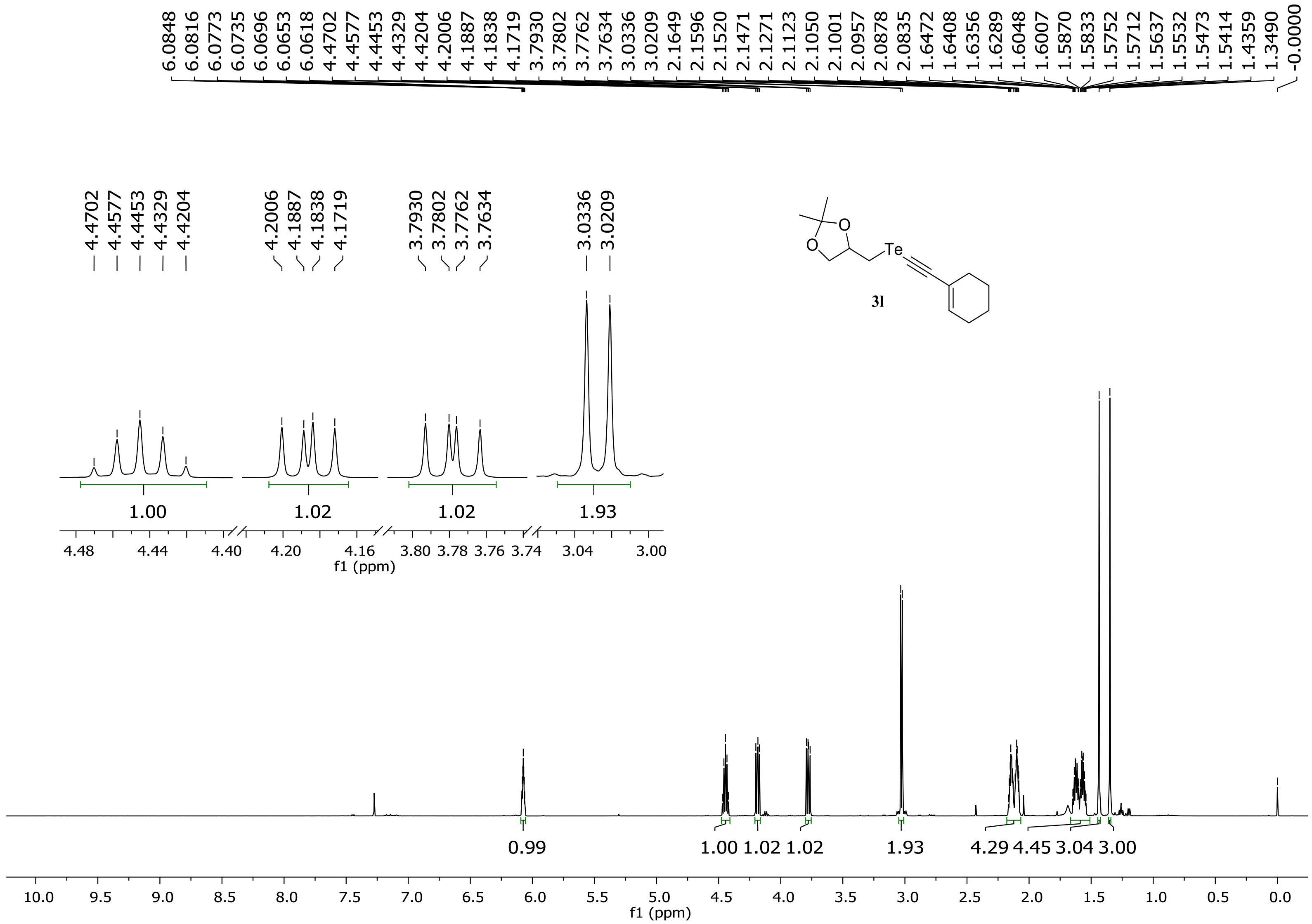

Figure S15. ^{1}H NMR ($CDCl_3$, 500 MHz) of the product **3l**.

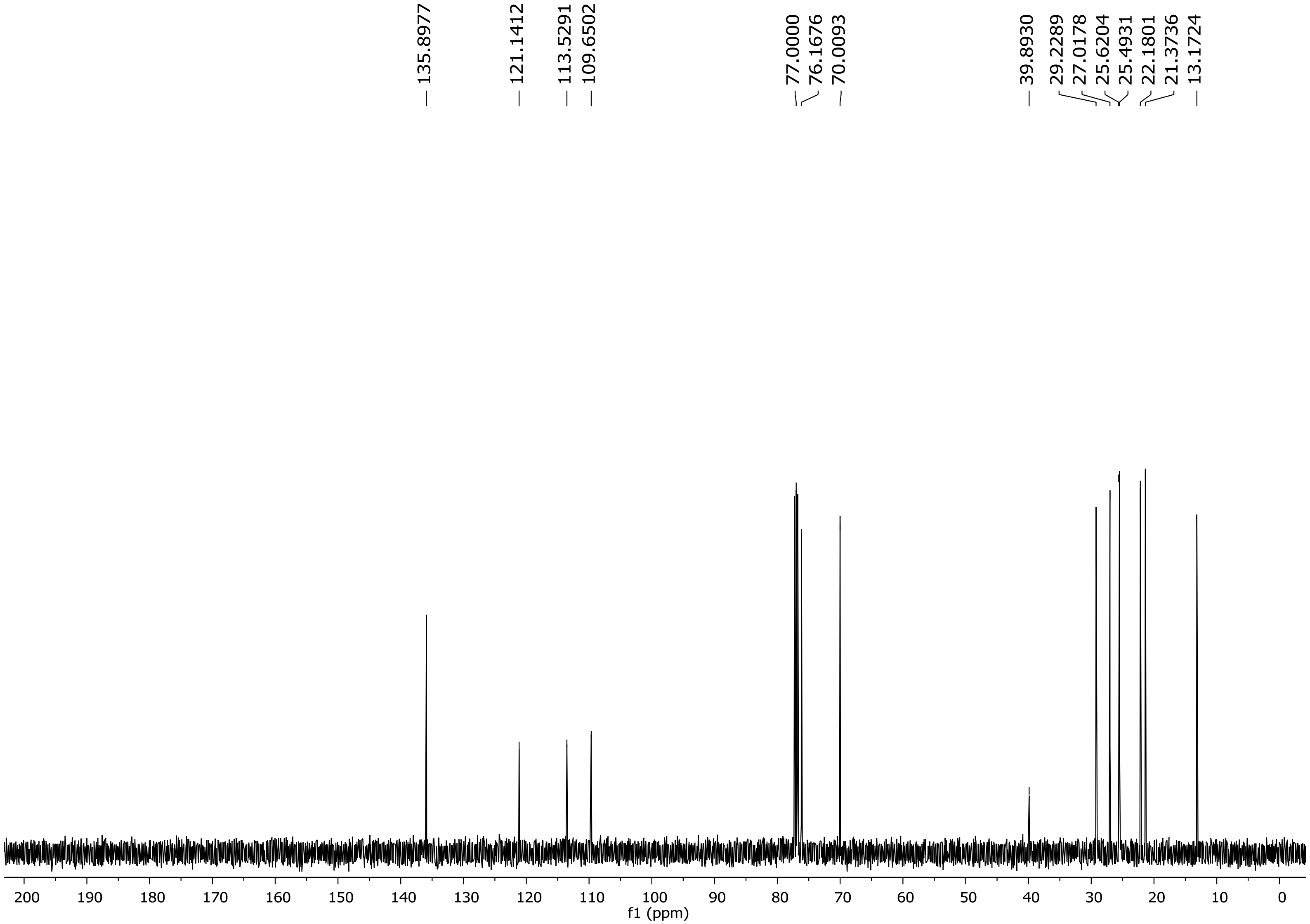

Figure S16. ^{13}C NMR ($CDCl_3$, 125 MHz) of the product **3l**.

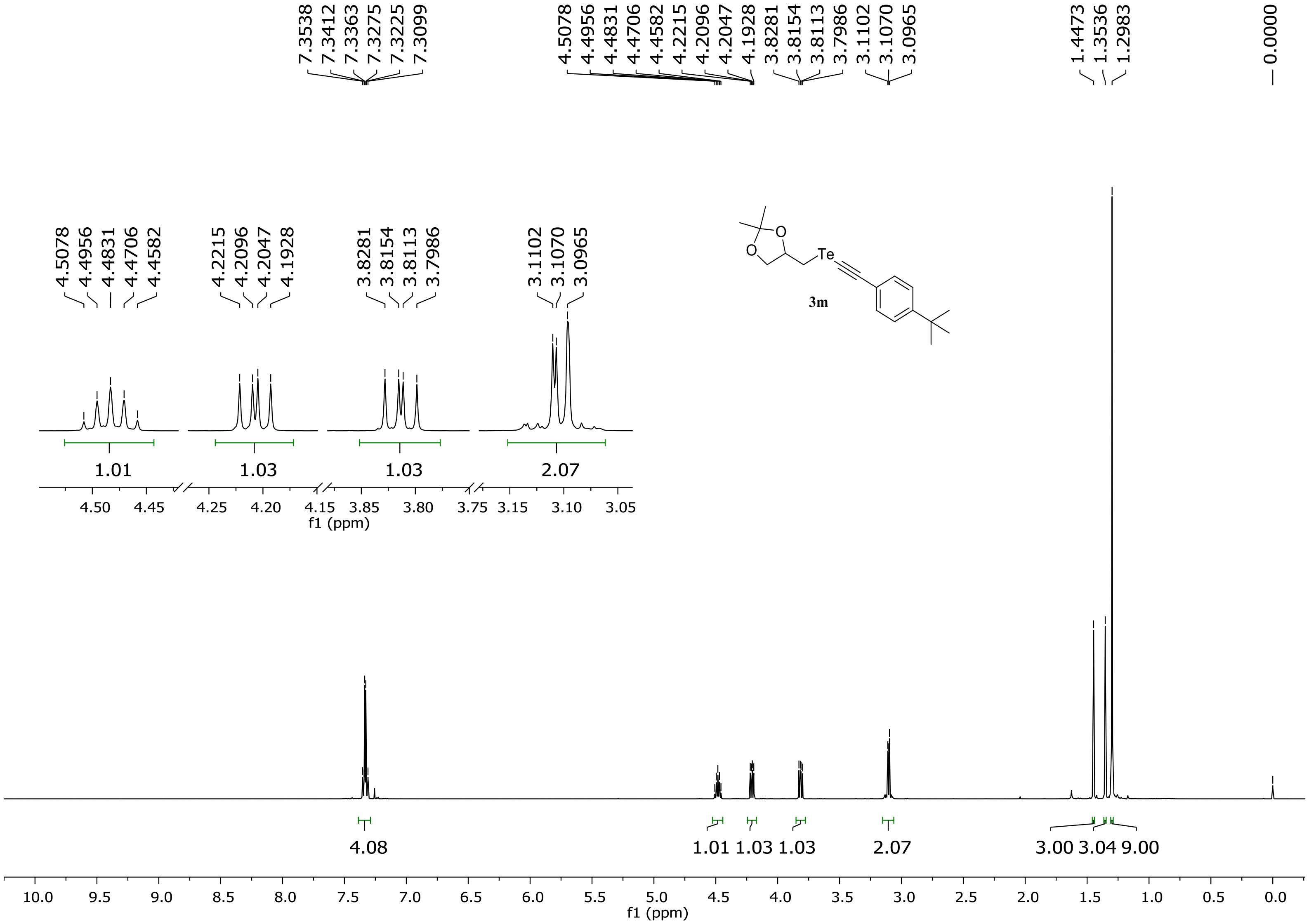

Figure S17. ^{1}H NMR ($CDCl_3$, 500 MHz) of the product **3m**.

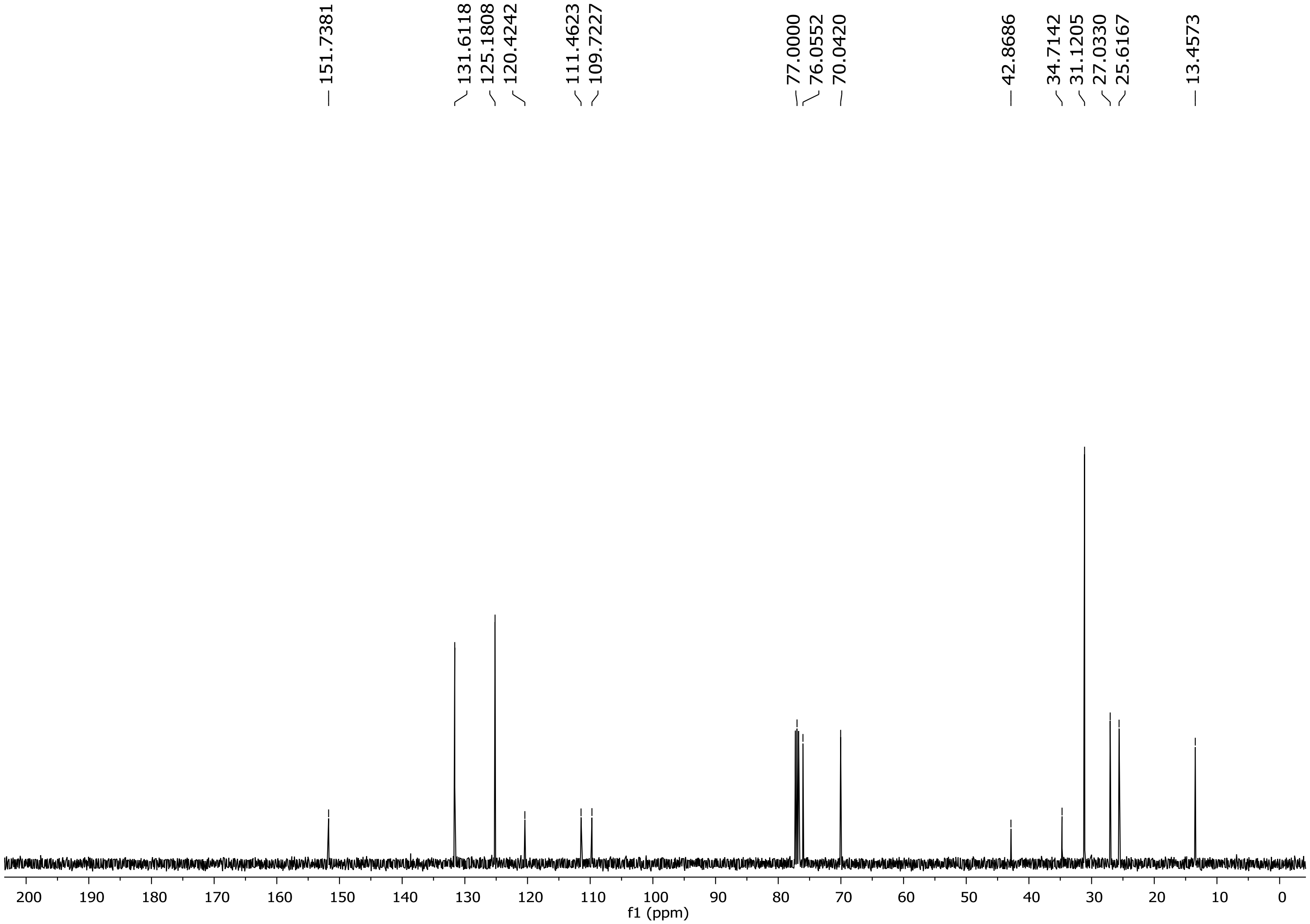

Figure S18. ^{13}C NMR ($CDCl_3$, 125 MHz) of the product **3m**.

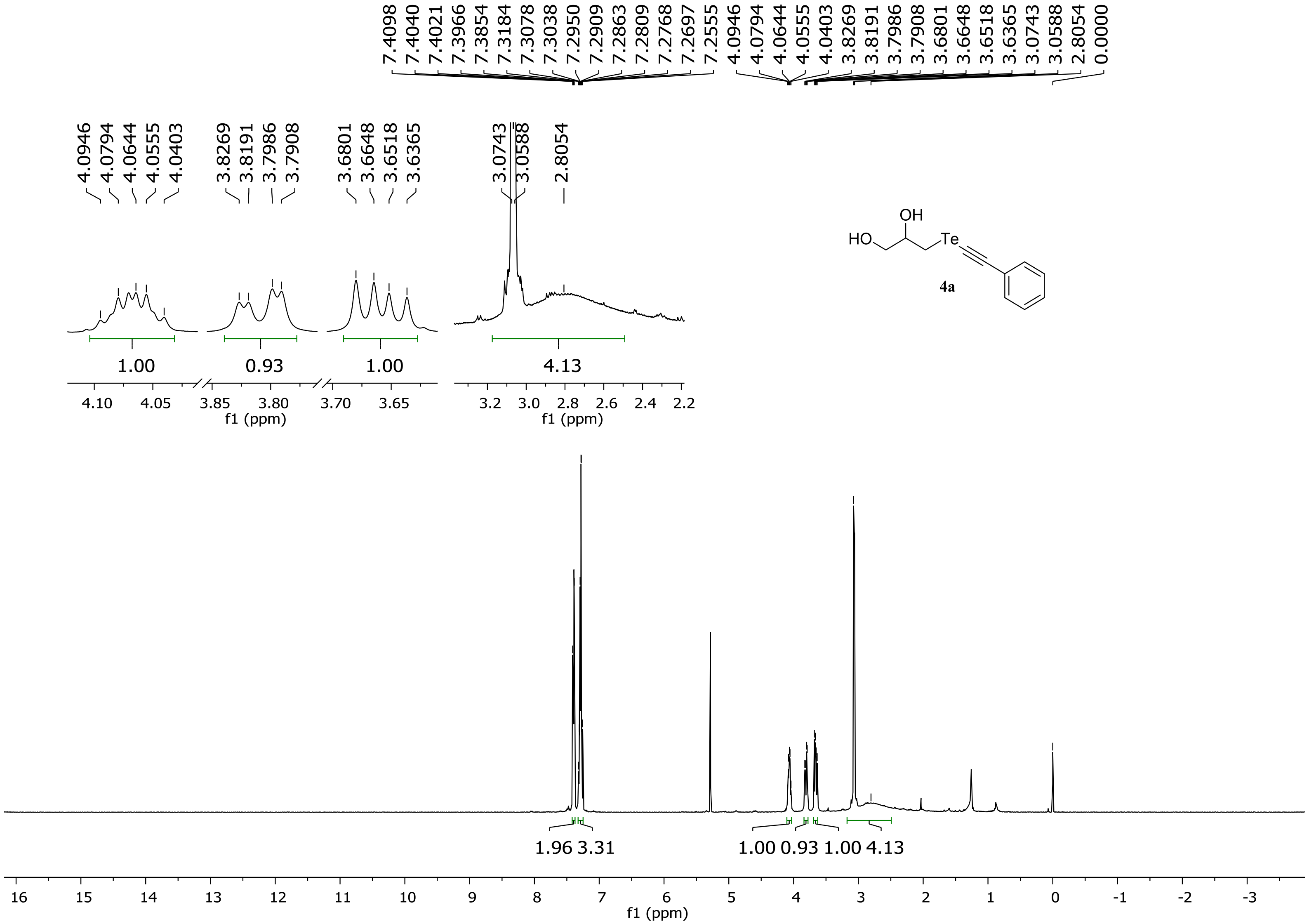

Figure S19. ^{1}H NMR ($CDCl_3$, 400 MHz) of the product **4a**.

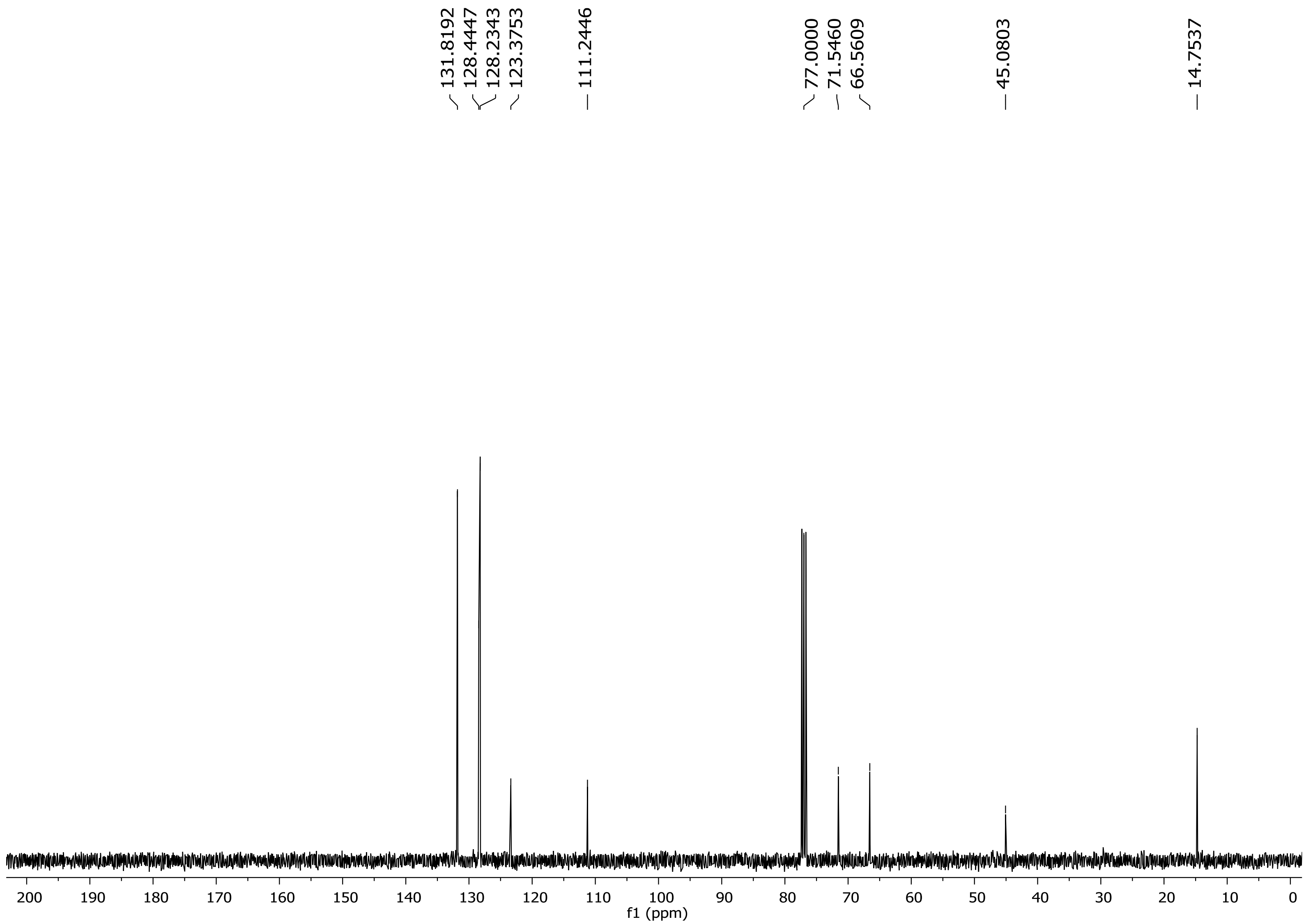

Figure S20. ^{13}C NMR ($CDCl_3$, 100 MHz) of the product **4a**.

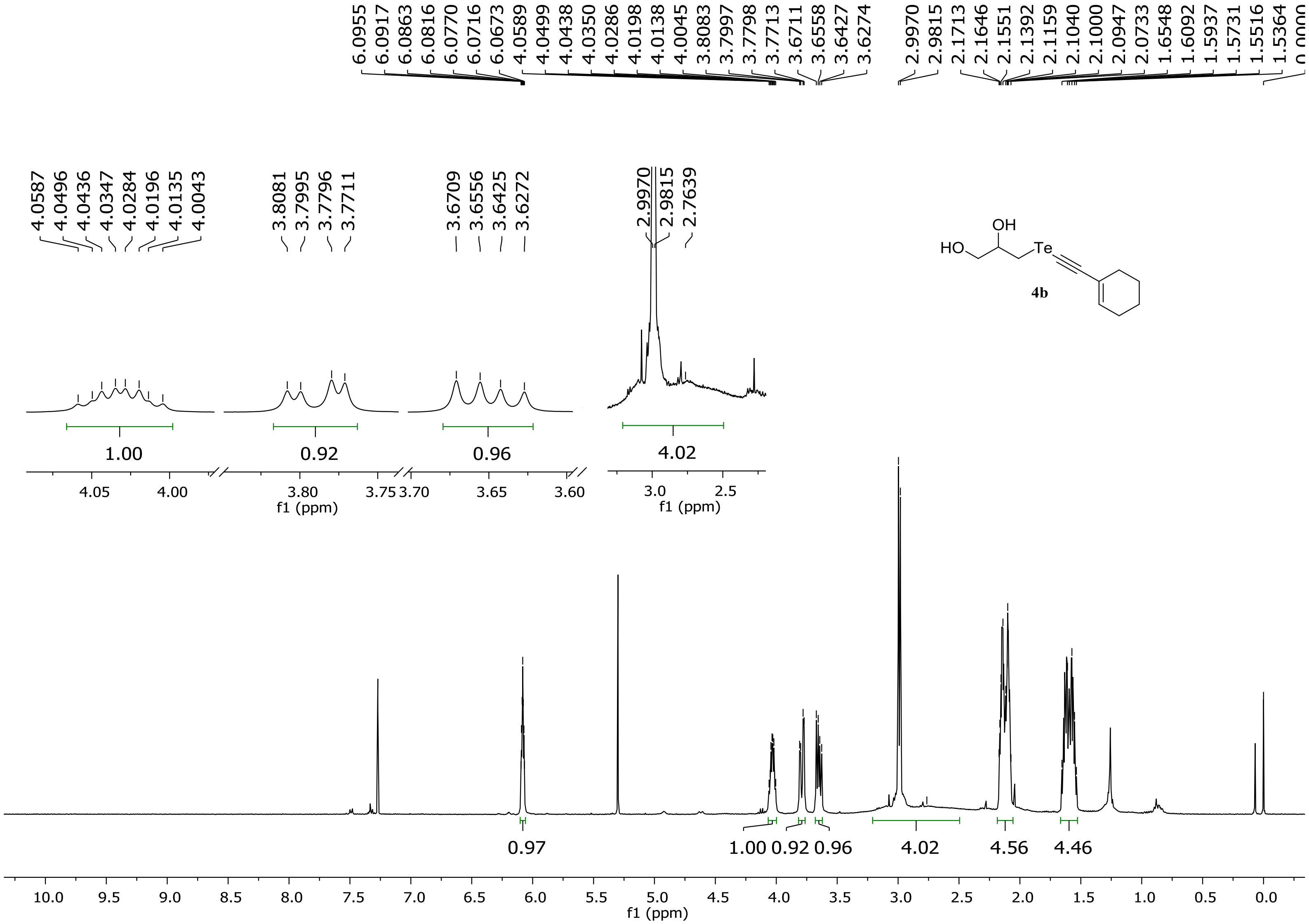

Figure S21. ^{1}H NMR ($CDCl_3$, 400 MHz) of the product **4b**.

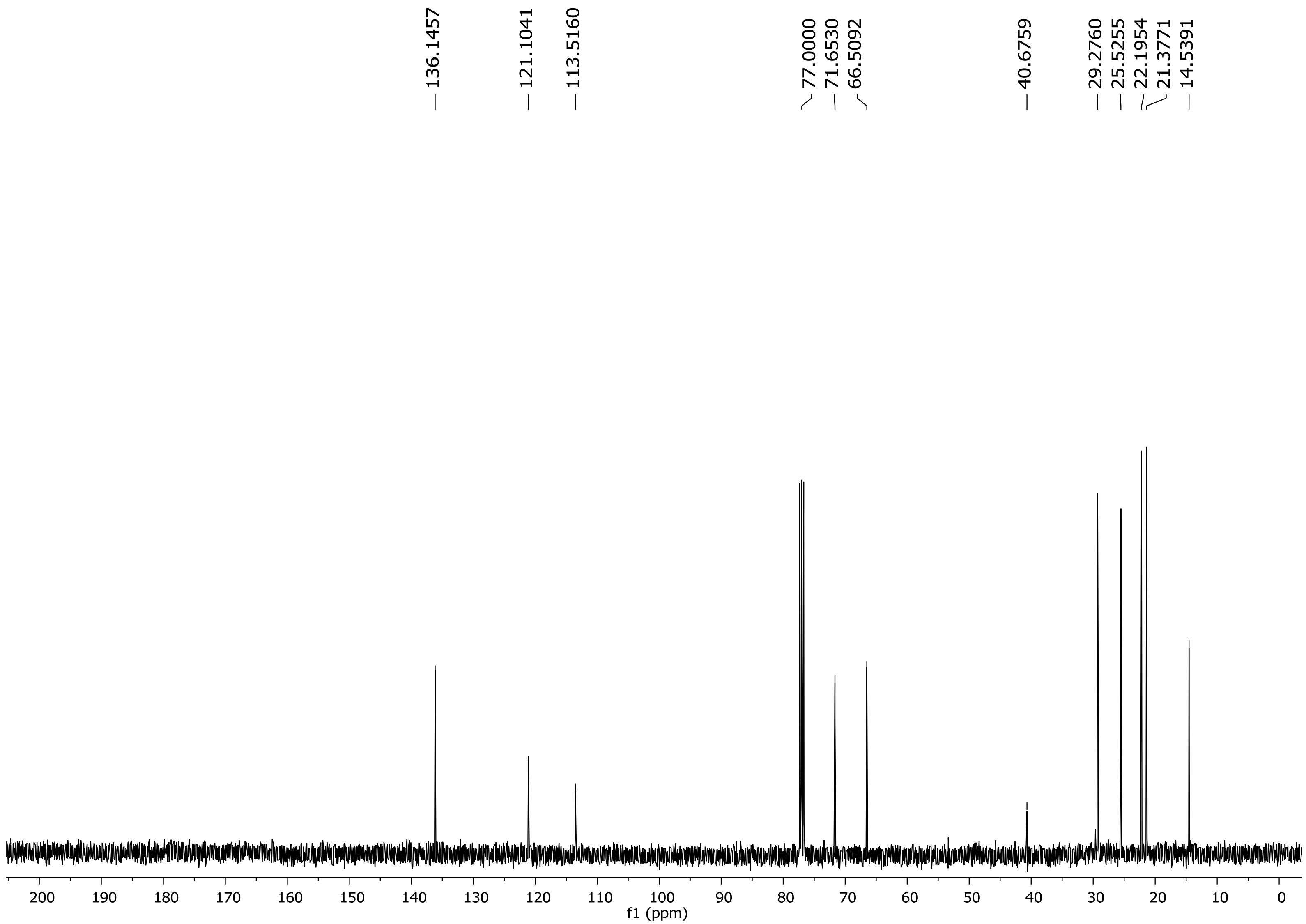

Figure S22. ^{13}C NMR ($CDCl_3$, 100 MHz) of the product **4b**.

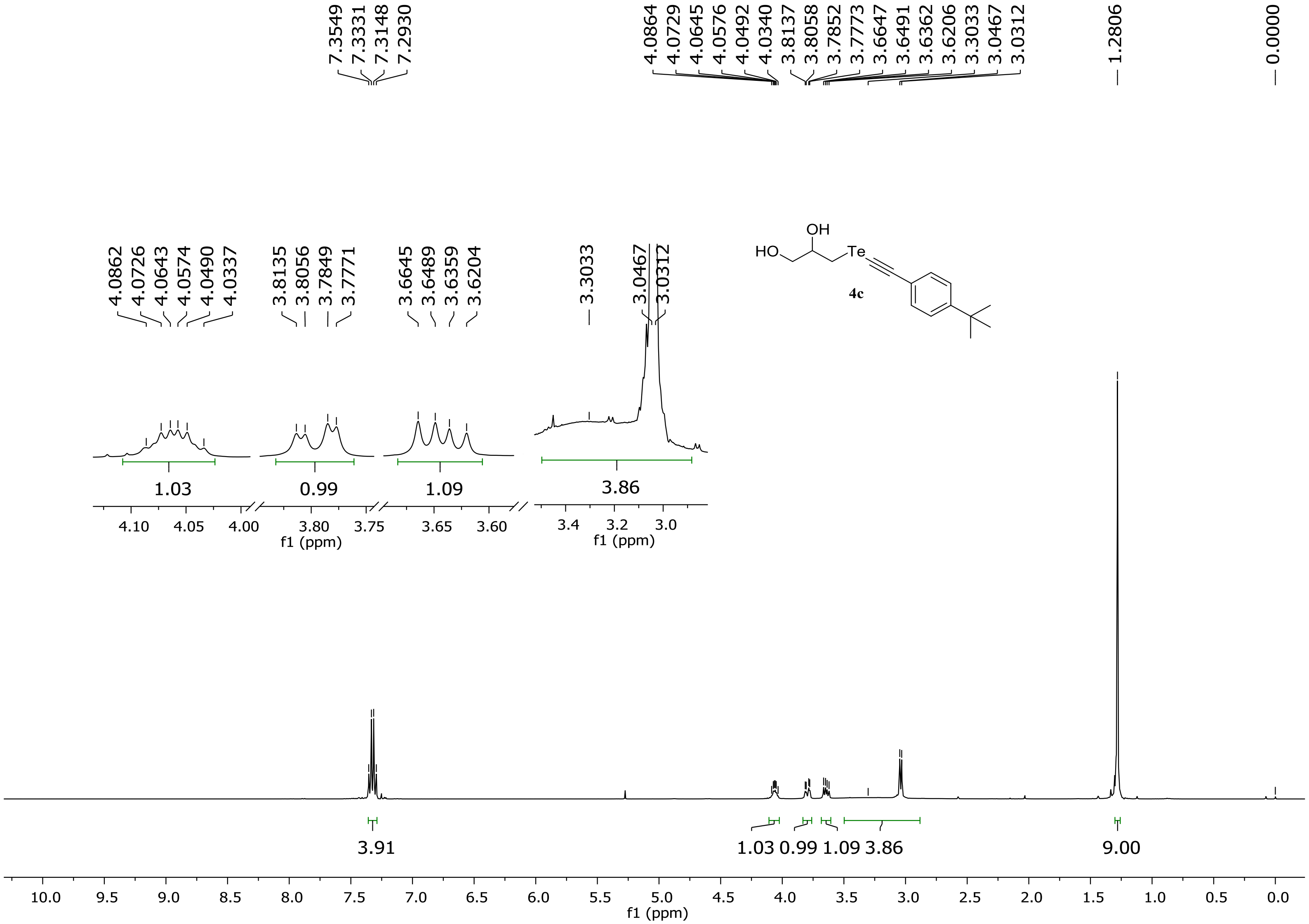

Figure S23. ^{1}H NMR ($CDCl_3$, 400 MHz) of the product **4c**.

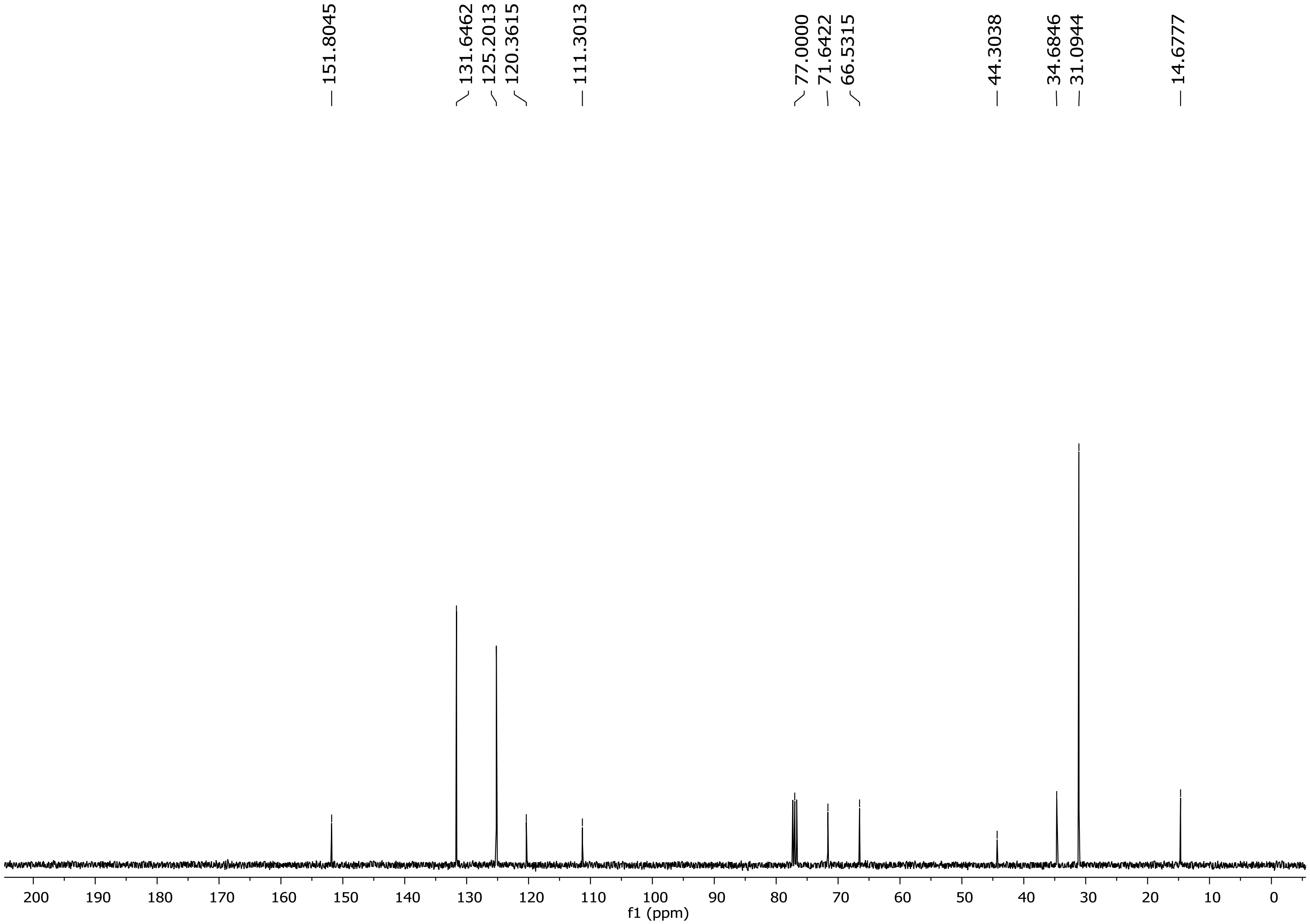

Figure S24. ^{13}C NMR ($CDCl_3$, 100 MHz) of the product **4c**.

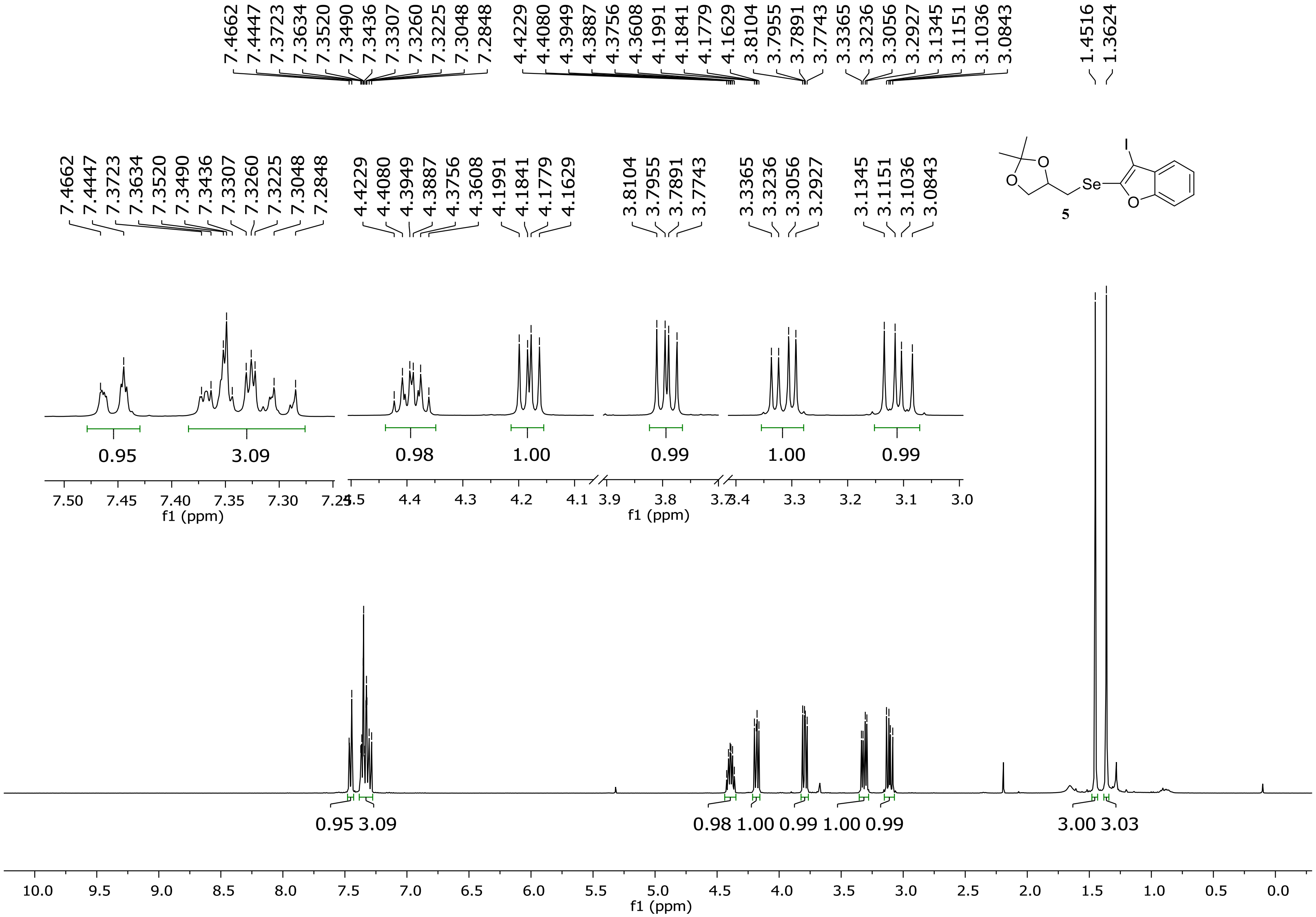

Figure S25. ^{1}H NMR ($CDCl_3$, 400 MHz) of the product **5**.

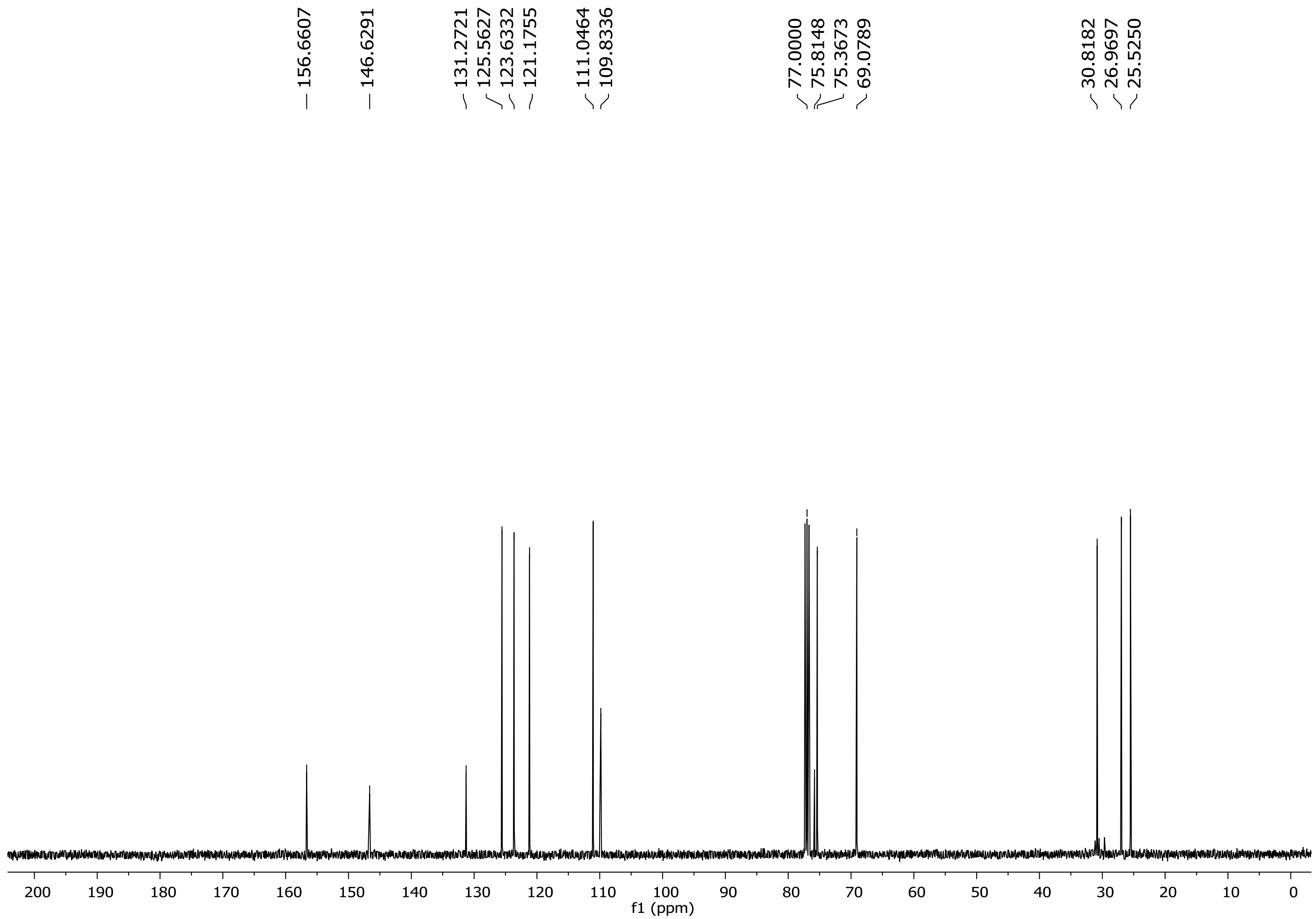

Figure S26. ^{13}C NMR ($CDCl_3$, 100 MHz) of the product **5**.
